# Phage-Antibiotic Combination Treatments: Antagonistic Impacts of Antibiotics on the Pharmacodynamics of Phage Therapy?

**DOI:** 10.3390/antibiotics8040182

**Published:** 2019-10-11

**Authors:** Stephen T. Abedon

**Affiliations:** Department of Microbiology, The Ohio State University, Mansfield, OH 44906, USA; abedon.1@osu.edu

**Keywords:** bactericidal, bacteriophage therapy, bacteriolytic, phage productive, phage therapy, productive infection, virion productive

## Abstract

Bacteria can evolve resistance to antibiotics. Even without changing genetically, bacteria also can display tolerance to antibiotic treatments. Many antibiotics are also broadly acting, as can result in excessive modifications of body microbiomes. Particularly for antibiotics of last resort or in treating extremely ill patients, antibiotics furthermore can display excessive toxicities. Antibiotics nevertheless remain the standard of care for bacterial infections, and rightly so given their long track records of both antibacterial efficacy and infrequency of severe side effects. Antibiotics do not successfully cure all treated bacterial infections, however, thereby providing a utility to alternative antibacterial approaches. One such approach is the use of bacteriophages, the viruses of bacteria. This nearly 100-year-old bactericidal, anti-infection technology can be effective against antibiotic-resistant or -tolerant bacteria, including bacterial biofilms and persister cells. Ideally phages could be used in combination with standard antibiotics while retaining their anti-bacterial pharmacodynamic activity, this despite antibiotics interfering with aspects of bacterial metabolism that are also required for full phage infection activity. Here I examine the literature of pre-clinical phage-antibiotic combination treatments, with emphasis on antibiotic-susceptible bacterial targets. I review evidence of antibiotic interference with phage infection activity along with its converse: phage antibacterial functioning despite antibiotic presence.

## 1. Introduction

Bacteriophages, or phages, are the viruses of bacteria. A majority of bacteriophages display lytic infection cycles. These involve phages infecting bacteria lethally and, in the process, producing new bactericidal agents, i.e., new phage virions. Phages have been employed clinically to treat bacterial infections for roughly one-hundred years [1,2], and many of these efforts seem to have been successful, e.g., such as in terms of treatment of chronic infections [3,4], wound infections [4,5,6], or lung-associated infections [7,8,9,10]. This ‘phage therapy’ furthermore has been proven to be efficacious in at least one modern efficacy (phase I/II) clinical trial [11], and recently there have been several well publicized phage therapy case-study successes [12,13,14]; see also [4,9,10,15,16,17]. Phages also can be effective against bacterial biofilms, e.g., [18,19,20,21], as well as against persister cells [22,23], though phages do not necessarily actively kill bacteria while these cells are still in the low-growth persister state [24]. In general terms [25], phages in phage therapy represent an older category of antibacterial agents that just might be able to address current needs for new antibacterial agents.

Given this history, an important question arises: Why has phage therapy not caught on more broadly as a reasonable alternative to antibiotics, particularly in North America and much of Europe? This question is especially relevant in light of issues associated with standard antibiotic effectiveness, the need for new approaches to treatment of bacterial infections [26], and several advantages inherent to phage use as antibacterial agents [27,28,29,30,31,32]. I suggest that part of the answer to this question of why not more phage therapy is that insufficient consideration has been placed on integrating phages into standard-of-care treatments of bacterial infections, that is, in terms of developing phage treatments which are concurrent with antibiotic treatments. Recently, however, there has been renewed focus on phage-antibiotic co-treatments in terms of preclinical experimentation [33]. Indeed, including antibiotics in experimental phage treatments could be relevant for reasons of expediting regulatory approval for phage therapy [34], and phages have been used in combination with antibiotics in various published clinical case studies [10,12,13,15,16,17]. Furthermore, by employing phage-antibiotic combination therapies, a potential can exist—as combination therapies [35,36,37,38]—for interfering with bacterial evolution to both phages and antibiotics [39,40,41]. For consideration of the potential for bacteria to evolve resistance to phages alone, see References [42,43,44,45].

Antibiotics are expected to interfere with aspects of bacterial physiology that can be crucial to phage antibacterial activities, e.g., by interfering with bacterial ribosome functioning. Emphasis here therefore is on documenting the impact of antibiotics on phage-infection pharmacodynamic aspects, i.e., retention of phage ability to negatively impact targeted bacteria despite antibiotic co-treatment. These pharmacodynamic properties include a retention, by bacteria-infecting phages, of both gene expression and antibacterial activity, and also of associated in situ—within treated patients—phage virion production. I address in particular the question of whether or to what degree antibiotic treatments may be antagonistic to the activity of phage infections, and this is especially given treatments in which antibiotic concentrations are greater than or equal to MICs (minimum inhibitory concentrations) [46,47,48] for targeted, especially clinically antibiotic-susceptible bacteria. This emphasis is rather than on considerations of the impact of phage-antibiotic combination therapy on bacterial resistance evolution [41], of phage-antibiotic synergy (PAS) while employing sub-inhibitory (<1× MIC) antibiotic doses [49], explicitly of treatment-order effects such as dosing with phages prior to dosing with antibiotics [33], or with otherwise substantial discussion of phage-antibiotic facilitative, additive, or synergistic interactions [18]. For general overviews of the subject of phage-antibiotic combination treatments, particularly as they relate to phage therapy, see Torres-Barceló and Hotchberg [41] and Tagliaferri et al. [33].

A defining characteristic of the literature concerning phage infection activity in the presence of antibiotics, given bacteria presumptive exposure to ≥1× MIC of antibiotic, is that different studies tend to present only one of two more-or-less opposing perspectives. These are those studies providing evidence or at least a premise that antibiotics will tend to profoundly interfere with phage infection outputs, particularly interfere with phage virion production, versus studies which suggest instead that antibiotic treatment of bacteria will not substantially interfere with phage infection outputs, or at least not interfere to a point where phage antibacterial activity is excessively diminished. It is the evidence underlying the bases of these two contradictory pharmacodynamic, phage-associated viewpoints that I review here. I start by looking at evidence consistent with antibiotic interference with phage infection activities and then consider evidence instead for continued phage infection activity despite antibiotic presence. A tentative overall conclusion is that the more complex the treatment environment, such as especially as found in vivo, then potentially the more compatible that phage therapy may be with antibiotic treatment.

## 2. Some Preliminary, General Considerations

The bulk of this article serves as a review of pre-clinical experimentation in which phage treatment or phage infection of bacteria is combined with antibiotic treatment. Emphasis is on interpretation of studies especially regarding the potential for phages to display various activities during their infection of bacteria. These activities, in terms of positive phage pharmacodynamic impacts on bacteria, can be differentiated into bactericidal effects, bacteriolytic effects, and also virion production by phage-infected bacteria. Generally, virion production and subsequent release by lytic phages would imply phage-mediated bacterial lysis, which in turn would imply phage-mediated bactericidal effects. Assessing experiments for these effects, as they may occur in the presence of 1× MIC or greater concentrations of antibiotic, however is not always straightforward, as considered in this section.

Firstly, various experiments or controls are useful for assessing antibiotic impact on phage infection activities. First are determinations of MICs for bacterial hosts. Conditions used for testing antibiotic impact on phage infection activity ideally would be equivalent to those conditions used for MIC determinations in terms of use of the same medium, use of the same antibiotic-incubation time frames, use of the same bacterial densities, and use of bacteria found in the same physiological state, such as log- versus stationary-phase. Second is comparing phage infection activities during antibiotic treatments to phage infection activities as occur under antibiotic-minus conditions; such antibiotic-minus infections in principle should allow full phage infection activity. Therefore, phage-only, antibiotic-only, phage-plus-antibiotic, and neither phage nor antibiotic treatments ideally would represent the minimum number of comparisons provided by an experiment. Third, if possible, it can be helpful to compare experiments using antibiotic-susceptible bacteria to equivalent experiments involving antibiotic-resistant host mutants. Phage infections of such bacteria may also allow full phage infection activity.

Exposure of bacteria to an antibiotic to which a bacterium is susceptible can in principle block subsequent phage infection-mediated bacteria killing. This proposed blockage can be due for example to antibiotic-caused interference with phage gene expression. Either with or without phage-mediated bactericidal activity, however, bacteria in the presence of 1× MIC antibiotic should not produce either colonies or turbid cultures. This has the effect of making it difficult to assess phage infection-mediated bactericidal activity, versus bacteriolytic or phage production activities, particularly while phage-infected bacteria are still in the presence of antibiotic, i.e., as measured in terms of reductions in bacterial colony-forming units (CFUs). Phage bactericidal activity might instead be assessed by first diluting antibiotic away from bacteria so that they may produce colonies if still able to, as following phage adsorption, but this would not be proof that phage-mediated bactericidal activity would also be retained while phage infections are still in the presence of antibiotic.

Phage-associated anti-biofilm activity can occur also as a consequence of the presence of biofilm-matrix degrading enzymes [50,51]. Such enzymes can be associated with phage virions and could mimic phage bactericidal activity in the presence of biofilms. That is, reductions in biofilm presence, e.g., in terms of reductions in biofilm biomass or reductions in numbers of constituent bacteria, can occur following phage treatment even without phages actually killing those bacteria. Such issues can arise given assays for phage activity in the presence of antibiotics when such assays are based solely on reductions in biofilm presence, particularly when applying phages at relatively high multiplicity of infections (MOIs; e.g., MOI = 5 or 10) and given biofilm washing prior to the quantification of anti-biofilm effects (i.e., so as to remove biofilm-released CFUs prior to their enumeration). Therefore, simply phage-associated removal of existing biofilm, especially given phage display of biofilm-matrix degrading enzymes, is not always indicative of phage infection bactericidal activity in the presence of an antibiotic.

Delays can exist between antibiotic application and the point of full antibiotic impact on bacterial metabolism, thereby potentially allowing for phage infection activity prior to full antibiotic impact on bacteria. Experiments in which phages and antibiotics are applied simultaneously or in which antibiotics are applied minutes after phage application therefore might provide a false sense of the potential for or degree of phage infection activity in the presence of an antibiotic. In addition, not all antibiotics are rapidly acting, in terms of degrading bacterial metabolic activity, e.g., such as cell-wall synthesis-inhibiting antibiotics for which duration of exposure along with antibiotic concentration are important components of their impact (versus concentration as a sole, key determinant). Phage infection activity thus in principle could occur during experiments prior to substantial antibiotic impact on bacterial functioning.

Antibiotic concentrations can decline over the course of experiments, particularly given long (multi-day) treatments and application of only a single antibiotic dose [47,48]. Such antibiotic decay could allow for phage infection activity that occurs later during experiments rather than earlier, and thereby phage infection activity that is not necessarily concurrent with ≥1× MIC antibiotic concentrations. In addition, phage infection activity if present might not be associated with the same individual bacteria that are substantially affected by antibiotic, such as due to heterogeneity in antibiotic impact on bacterial cultures as might be observed in association with bacterial biofilms. Consider also the occurrence of antibiotic tolerance as can be associated with bacterial growth in biofilms [47,48,52,53,54,55,56]. In addition, given sufficient lengths of treatment, and sufficiently low starting phage numbers (MOI < 1), then substantial phage virion production could occur during infection of antibiotic-*resistant* bacteria, that is, so long as those bacteria have first been allowed to grow to relatively high densities. Any of these scenarios could result in a false impression of occurrence of phage infection activity, such as virion production in the presence of even seemingly substantially antibiotic-impacted populations of antibiotic-susceptible bacteria. 

Generally, it also is important to be aware that there is a difference between phage activity as occurs immediately upon infection (i.e., pharmacodynamic effects) versus bacterial resistance evolution as occurs over longer time frames. Not all experimental treatments, particularly ones of long duration and that are assessed only in terms of changes in relative bacterial presence, will necessarily distinguish between these two phenomena. Resistant bacteria, especially given phage and antibiotic treatments of long duration, can by their presence mimic reduced bactericidal activity by individual agents as relative to phage-antibiotic combined treatments (that is, more bacteria may be present after, e.g., two days of incubation given antibiotic-only treatment than with phage-plus-antibiotic treatment, but possibly only because the latter inhibits bacterial resistance evolution better than the former). This potentially false negative phage-mediated bactericidal activity would be due to different likelihoods of bacterial mutation in combination with resistant-bacterium grow back given application to cultures of single versus multiple antibacterial agents. Comparisons of bacterial numbers following, e.g., 24 or more hours of treatment, thus may be less indicative of phage bactericidal activity in the presence of antibiotic than somewhat sooner assessments, i.e., as made prior to substantial resistant-mutant bacterial grow back.

Lastly, phage virion production can be inferred, within more complex experimental systems such as those consisting of biofilms, from measurements of bacteria-killing activity. Doing so requires knowledge of initial ratios of added phages to bacterial numbers and is based on Poisson distributions [57]. For example, an MOI of 1 without additional phage population growth would be expected to kill at most 1 – e^−MOI^ = 1 – e^−1^ = 63% of bacteria present. Therefore, any bacteria killing that is in excess of 63%, particularly killing that is attributable to phage action, could in this example be indicative of excessive virion numbers and thereby of in situ phage virion production following dosing [58]. The likelihood that virion production has occurred as based on such analyses would be greater the lower the starting phage MOI and the greater the difference, in terms of bacterial removal, between antibiotic-only treatment and phage-plus-antibiotic treatment. 

## 3. Antibiotic Interference with Phage Pharmacodynamics

Antibacterial agents can be distinguished into those which are bacteriostatic versus those which are bactericidal [59]. A general expectation is that bacteriostatic agents may interfere with the action of bactericidal agents [60,61], and this is so particularly to the extent that the latter are more effective against fully metabolically active bacteria rather than bacteria which are less metabolically active [62,63]. The same situation may be present with phages, also as bactericidal agents, as phages too tend to display less activity given reduced bacterial metabolic activity [64,65,66]. A difference, however, is that individual phage types can generate multiple activities in association with target bacteria. Depending on circumstances, these activities can range from phage gene expression, to bactericidal or bacteriolytic activities, to production of new phage virions, or indeed and typically all of the above. 

That antibiotics might interfere with phage infection activity is not a new idea, e.g., as documented by Adams [67]. Indeed, there are many bacterial components that are essential for bacteria to function and essential also for phage infections to function. The most obvious but not only such component is the bacterial ribosome, against which several antibiotics act, e.g., clindamycin, erythromycin, linezolid, and tetracycline. In considering the potential for phage therapy to work in the course of co-treatments with antibiotics, against antibiotic-susceptible bacteria, it therefore is critical to question to what degree antibiotics used in association with phages might interfere especially with phage bactericidal activity, and associated virion production activities. The retention of phage functionality furthermore should be tested at antibiotic concentrations that meet or exceed antibiotic MICs for targeted bacteria, as these are the antibiotic levels that are both expected and desired in vivo during antibiotic treatments. Our expectation, on the other hand, is that sub-MIC antibiotic concentrations may not interfere with phage antibacterial activity. Such continued functioning in the presence of sub-inhibitory antibiotic concentrations in fact represents a central tenet of the PAS literature [33,49]. As noted, however, PAS as a phenomenon is not being considered in detail here.

By way of example, regarding antibiotic interference with phage infection activity, Matsui et al. [68] describe four phages of *Ralstonia solanacearum*—ΦRSF1, ΦRSL1, ΦRSL2, and ΦRSB1—for which phage virion production is fully blocked given bacterial exposure to 5 μg/mL rifampin (20 min incubation prior to phage infection). Here the calculated MIC for this host was 3 μg/mL, and thus phage virion-production was inhibited given exposure to ~2× MIC. Furthermore, as Tagliaferri et al. [33] suggest (p. 10), “Negative interference [of antibiotics with phages] might be more common [than] as assumed, and it is possible that such experimental outcomes in the laboratory are less frequently reported than the positive ones.” Notwithstanding this Tagliaferri et al. concern, a great deal of published evidence is consistent with the Matsui et al. result, particularly involving *Mycobacterium tuberculosis* as host. In this section, I summarize experiments indicating a potential for antibiotics to interfere with such phage infection activities. As follows, CFU stands for Colony-Forming Units and PFU stands for Plaque-Forming Units. As throughout this review, I both summarize the results of individual studies and provide my interpretations of those results, presenting the material in ascending author-date order. See Table 1 for summary of the studies presented in this section.

### 3.1. Mycobacterium tuberculosis Studies

*M. tuberculosis* is both an important human pathogen and an extremely slow growing bacterium. As a consequence of the latter property, diagnosis of antibiotic resistance in *M. tuberculosis* can take far longer than can be clinically desirable. To reduce the duration of this process, such as down to on the order of a few days, a number of groups have explored the use of phages as indicators of *M. tuberculosis* metabolic activity in the presence of antibiotics. Here reduced bacterial metabolic activity, as a consequence of antibiotic exposure, is assumed to correlate with reduced phage infection activity, activity as measured by various means. In this subsection I review a sampling of this literature, which mostly is supportive of and indeed premised upon the idea that antibiotics can be antagonistic to phage infection activities.

#### 3.1.1. Jacobs et al., 1993, *Mycobacterium tuberculosis*, Various Antibiotics

A luciferase-encoding reporter phage was developed by Jacobs et al. [69], based on the *Mycobacterium smegmatis* phage TM4, with a goal of assessing gene expression during infection of *M. tuberculosis* in the presence of antibiotic. They found that after 48 h of antibiotic incubation, luciferase expression is knocked out when the treated bacteria are antibiotic susceptible but not when bacteria instead are antibiotic resistant. In this case, the antibiotics were isoniazid (1 μg/mL), rifampin (2 μg/mL), or streptomycin (6 μg/mL). As this was a luciferase-based test, light output would be dependent not just on reporter-gene expression following phage infection but also on ATP levels within the infected bacteria, levels of both which should positively correlate with phage infection activity (i.e., more phage gene expression and more intracellular ATP should both result in greater phage infection activity). A multiplicity of 1000 was employed and 80-fold greater luminescence was reported without either rifampin (2 μg/mL) or streptomycin (6 μg/mL) present, and ten-fold greater without isoniazid (1 μg/mL), thus indicating substantial inhibitions of phage infection activity by these antibiotics. These results were not correlated in this study with MICs, however.

#### 3.1.2. Wilson et al., 1997, *Mycobacterium tuberculosis*, Isoniazid and Rifampin

Wilson et al. [70] used phage D29 to measure virion production in the presence and absence of antibiotic while infecting *M. tuberculosis*. Phage plaque formation after termination of antibiotic treatment was used as an indicator of phage virion production, with *Mycobacterium smegmatis* serving as the plaquing indicator bacterial strain. Antibiotic treatments prior to phage addition (~10 ^9^ PFU/mL in situ concentration) consisted of three days of incubation in the presence of isoniazid at concentrations of 2 μg/mL or rifampin at concentrations of 4 μg/mL (resistance was defined as an ability to grow in the presence of 2 μg/mL or more and 1 μg/mL or more, respectively). Generally, plaque formation was associated, as expected, with bacterial antibiotic resistance while no plaque formation was seen given antibiotic sensitivity, as was found to be true for 44 of 46 isolates tested. The two outlying *M. tuberculosis* isolates were reportedly rifampin-susceptible but nevertheless able to support phage virion production despite the antibiotic’s presence. Equivalent concordance of potential to support plaque formation, and isoniazid resistance versus sensitivity, was seen for 40 of 46 isolates tested: two otherwise resistant strains failed to support plaque growth in the presence of isoniazid, while four susceptible isolates did support plaque growth. Generally, then, prior antibiotic treatment was found to interfere with the ability of antibiotic-susceptible but not antibiotic-resistant bacteria to support the production of at least one phage virion, though with some exceptions. Of possible relevance to these exceptions, standard MIC determinations were done over one- to two-week periods while antibiotic treatment prior to phage infection was for a total of three days.

#### 3.1.3. Galí et al., 2003, *Mycobacterium tuberculosis*, Rifampin

Galí et al. [71] used microtiter plates to treat *M. tuberculosis* isolates with rifampin (5 μg/mL) and then with phage D29 (10^7^ PFU/mL). Bacteria were incubated in the presence of antibiotic for 24 h and then in the presence of phages for 90 min at 37 °C. After inactivation of unadsorbed phages, the production of new phages was detected via spot assays (application of 10 μL of potentially phage-containing culture to a growing lawn of *M. smegmatis* [86]). In the presence of rifampin, all rifampin-resistant *M. tuberculosis* isolates supported phage virion production whereas none of the rifampin-susceptible isolates did. This is an indication that antibiotic presence interfered with the ability of antibiotic-susceptible bacteria to support phage virion production. A rifampin-negative, spotting-positive control was performed for each bacterial isolate tested to rule out phage failure to infect independent of rifampin presence. MICs were not reported. See McNerney [87] for a formal protocol of this approach.

#### 3.1.4. McNerney et al., 2007, *Mycobacterium tuberculosis*, Rifampin

McNerney et al. [72] developed a colorimetric, microtiter plate-based approach using 3-(4,5-dimethylthiazol-2-yl)-2,5-diphenyltetrazolium bromide (MTT) dye towards assaying the impact of *M. tuberculosis* exposure to rifampin on phage D29 virion production. In this assay, colorimetrically indicated *M. smegmatis* culture-clearing (in the absence of antibiotic) was the indicator that phage virion-production had previously occurred in association with overnight rifampin-treated *M. tuberculosis*. This broth-based *M. smegmatis* clearing was compared with a plaquing-based determination of virion production, also post antibiotic exposure. In a small fraction of cases, phage virion production was seen given 2 μg/mL rifampin treatment of *M. tuberculosis* but not at 10 μg/mL. In most cases those bacterial strains deemed rifampin-susceptible, as based on genome sequence information, gave rise to blocked phage virion production even at the lower rifampin density (at least 70 out of 85). In one case, however, phage virion production was seen even at the higher rifampin concentration. As with all of the studies so far described, results predominantly were consistent with a lack of phage infection activity given bacteria exposure to antibiotic. MIC for *M. tuberculosis* strains were not reported.

#### 3.1.5. Pholwat et al., 2012, *Mycobacterium tuberculosis*, Various Antibiotics

Pholwat et al. [73] used qPCR to detect phage D29 virion production given *M. tuberculosis* exposure to several different antibiotics: amikacin, capreomycin, cycloserine, ethambutol, ethionamide, isoniazid, kanamycin, linezolid, moxifloxacin, ofloxacin, para-aminosalicylic acid, rifampin, and streptomycin. As part of this analysis, it was found that treatment with rifampin for 3, 6, 24, or 48 h prior to 24 h of phage treatment yielded substantially less phage signal with rifampin-susceptible versus rifampin-resistant bacteria. Equivalent treatment with isoniazid by contrast revealed a delay in impact. That is, 3- and 6-hour pre-incubations yielded similar phage signal as seen with a multidrug-resistant control, but by 24 and 48 h of antibiotic pre-incubation there was substantially less phage signal.

The difference in the impacts of the two drugs is accounted for by differences in their modes of action, with one acting bacterial cell-internally—where drug concentrations tend to be particularly relevant (rifampin inhibits RNA polymerase)—and the other acting by inhibiting cell-wall synthesis, where duration of drug exposure is also a key variable (in addition, bacteria potentially may have been dead before they were reached by phages after 24 or 48 h of antibiotic pre-incubation). Nevertheless, and contrary to the rest of the results reviewed in this section, this observation of delayed effects with isoniazid is consistent with phage production despite substantial antibiotic pre-treatment of susceptible bacteria, which in turn is suggestive that not all combinations of phage types, antibiotic types, antibiotic concentrations, and durations of pre-incubation will necessarily result in blocks on phage antibacterial activity, even if MICs are exceeded.

The antibiotic concentrations used were those provided by the World Health Organization as “critical concentrations”, that is, the amount required to observe therapeutic effects in patients given treatment of drug-susceptible bacteria. Experiments with all of the above-listed antibiotics—given 48-h pre-incubation and 24-h phage exposure—were consistent with a strong concordance between qPCR-based predictions of phage propagation and non-phage-based determinations of drug sensitivity versus resistance (concordance was mostly 100% but was 97% for capreomycin and 82% for ethionamide). These data, as with those seen in previous studies and except given insufficient duration of pre-incubation with isoniazid, are consistent with antibiotic-associated inhibition of phage virion production.

#### 3.1.6. Summary of Studies of *Mycobacterium tuberculosis* Phages

Overall, exploration of inhibition of phage infection activity in the presence of antibiotic, particularly employing the antibiotic, rifampin, has been extensive using *M. tuberculosis* as a host. Indeed, in 2010 a meta-analysis based on 31 different studies was published on the subject by Minion and Pai [88]. Limitations of these various studies to the question of whether antibiotic treatment can generally interfere with phage infection activity, however, is their use of *M. tuberculosis* as phage hosts versus additional bacterial types, relatively limited phage choice, and also questions concerning bacterial viability following long pre-incubations with antibiotic. Especially the first two of these limitations are addressed in the following two sub-sections.

### 3.2. RNA Polymerase Inhibitors and Non-Mycobacterium Phages

Because rifampin is an inhibitor of host RNA polymerase, any phages which carry their own RNA polymerase within their virions, deliver this enzyme upon adsorption, and otherwise do not require their host’s RNA polymerase during infections may not be susceptible to rifampin treatments of bacteria. Sensitivity would be in terms of rifampin inhibiting virion production ability, and phage resistance would be seen even when host bacteria are rifampin susceptible. In particular, phage φKZ of *Pseudomonas aeruginosa* is able to replicate, producing virions more or less normally in the presence of 400 μg/mL of rifampin, which is a concentration of rifampin that completely inhibits the growth of a different phage, LUZ19 [74]. Phage φKZ also possesses putative phage-encoding RNA polymerase genes and seems to lack host promoter sequences. With *Bacillus subtilis* phage PBS2, equivalent results were seen with rifampin treatment (20 or 100 μg/mL added 2 or 5 min prior to phage infection; MIC ≤ 1 μg/mL) and also with host RNA polymerase-inhibiting streptolydigin and streptovaricin, but not with the DNA-binding, RNA-synthesis inhibiting actinomycin D and lucanthone antibiotics [89]. Missing from these studies, however, are robust indications of the sensitivity of uninfected host bacteria to the same levels of antibiotic as applied to phage infections, i.e., they are lacking formal determinations of MICs. Numerous other phages have been found instead to be susceptible to these various RNA polymerase-inhibiting antibiotics, rifampin, rifamycin, or streptovaricin, including at MIC-level concentrations. These latter phages are considered in this subsection and to a degree in the next section as well. Although several studies explore the impact of these various drugs on RNA- or ssDNA-genomed phages, emphasis here is placed on dsDNA phages.

#### 3.2.1. Geiduschek and Sklar, 1969, *Bacillus subtilis*, Rifamycin

Geiduschek and Sklar [75] found that phage SPO1 of *B. subtilis* fails to lyse or produce new virion particles given exposure to 1 μg/mL rifamycin starting eight min prior to phage addition (MOI = 5). Both lysis and new virion production occurred more or less normally under the same conditions given infection of rifamycin-resistant mutants of the same host. Under these same conditions, the host was found to be unable to synthesize RNA transcripts as measured via incorporation of ^3^H uridine. Phage replication also could be blocked given a 2-min but not a 6-min delay between phage adsorption and rifamycin addition (phage addition followed by antibiotic addition), implying that sufficient transcription of phage genes had occurred by 6 minutes to allow the phage to carry out the rest of its life cycle. Phage replication thus is highly impacted by levels of rifamycin which also block host RNA synthesis. MICs for either rifamycin-susceptible or -resistant hosts, however, are not provided.

#### 3.2.2. Hemphill et al., 1969, *Bacillus subtilis*, Rifampin

Hemphill et al. [76] looked at burst sizes by phage β22 of *B. subtilis*. These phages were more or less fully inhibited by rifampin (10 μg/mL) when added sooner after the start of phage infection (MOI = 4) but only partially inhibited by rifampin when added later after the start of phage infection. Burst sizes were less than 1% of those without rifampin addition but increased substantially given rifampin addition at 35 min into infection cycles. MICs were not reported.

#### 3.2.3. Takeda et al., 1969, *Escherichia coli*, Rifampin

Takeda et al. [77] explored the potential for rifampin to interfere with phage λvir infection activity (MOI = 5). *E. coli* was treated with 25 μg/mL rifampin. With a rifampin-susceptible host, “phage growth” was reported as being “<10% of normal” in the presence of rifampin, while “normal” phage growth occurred in rifampin’s absence. Of interest, two hosts which are reported as displaying slight growth given exposure to this concentration of rifampin also are indicated as allowing “±” phage growth (“20–50% of normal”). Although not explicitly indicated, I assume that it is phage burst size that is being measured as “phage growth”, indicated as determined via single-step growth experiments [90,91], with ± phage growth also associated with “somewhat” delayed lysis. RNA synthesis during phage infection also dramatically declined following addition of 50 μg/mL rifampin 15 min into the infection cycle. These results are indicative of interference by rifampin with phage infection activity. They also indicate intermediate levels of interference in association with intermediate rifampin impact on the replication of host bacteria. The latter is suggestive of these antibiotic concentrations being lower than MIC, at least as approximated under the conditions employed (“slight… growth after overnight incubation”).

#### 3.2.4. Sokolova et al., 1970, *Escherichia coli*, Rifampin and Streptolydigin

Sokolova et al. [78] explored the impact of rifampin and streptolydigin on phage T2 infection (MOI << 1) of both antibiotic-susceptible and -resistant *E. coli*. Absent either drug, burst size was on the order of 200 in the doubly susceptible bacterium (rifampin and streptolydigin) but only 100 in the doubly resistant bacterium, suggesting existence of costs in phage infection activity associated with host antibiotic resistance. With susceptible bacteria, addition of either drug at 100 μg/mL, immediately after phage adsorption, in amounts which appear to exceed MICs, reduced burst sizes to negligible (1% of no treatment), whereas a 10-min delay in drug addition reduced burst sizes instead to 5% of no treatment.

#### 3.2.5. Beckman et al., 1972, *Escherichia coli*, Rifampin

Beckman et al. [79] determined the impact of rifampin (400 μg/mL, present from the start of phage adsorption) on *E. coli* phage T5 under single-step growth conditions. Burst sizes were reduced from 120 without rifampin down to zero with. Little impact was seen with rifampin-resistant hosts. MICs were not reported.

#### 3.2.6. Toussaint and Lecocq, 1975, *Escherichia coli*, Rifampin

Toussaint and Lecocq [80] induced phage Mu lysogens of *E. coli*, applied rifampin (100 μg/mL), and then assayed for phage virion production. This production was “blocked completely” with addition up to 15 min into the induced infection cycle. Inhibition of phage virion production was not observed, however, with a rifampin-resistant bacterial host. MIC for the *E. coli* host was not determined.

#### 3.2.7. Riveros-Moreno, 1975, *Pseudomonas* BAL-31, rifampin

Riveros-Moreno [81] employed phage PM2 (MOI = 10) of *Pseudomonas* BAL-31 and 0.3 μg/mL rifampin. Burst sizes were reduced to less than 1% given simultaneous (time 0) rifampin addition. The *Pseudomonas* BAL-31 MIC for rifampin is not indicated.

#### 3.2.8. Blasdel et al., 2017, *Pseudomonas aeruginosa*, rifampin

Blasdel et al. [82] found that 100 μg/mL rifampin, added 10 min prior to phages PAK_P3 or PAK_P4 (added at MOI = 1), blocked their “amplification”. MIC was not reported.

#### 3.2.9. Summary of Studies of RNA Polymerase Inhibitors and Non-*Mycobacterium* Phages

These various results are consistent with RNA polymerase-inhibiting antibiotics interfering with infections by different non-*M. tuberculosis* phages. Exceptional were certain phages whose virions appear to carry non-host, otherwise not antibiotic-susceptible RNA polymerases into newly infected bacteria. Otherwise, production of new virions often appears to be completely blocked in the presence of these various drugs. Unfortunately, however, it is difficult to correlate most of these phage infection-inhibition results, and associated antibiotic concentrations, with the MICs of host bacteria. Therefore, the major conclusion is that given sufficient concentrations of antibiotic, then phage infection activity can be blocked by rifampin, but it remains uncertain whether incubation, e.g., with 1× MIC, would result in equivalent interference. In addition, simply phage bactericidal activity was not measured in these experiments, also such as at 1× or greater MIC. That is, it is conceivable that phage antibacterial activities could be retained even at antibiotic concentrations that nevertheless are able to interfere with phage virion production.

### 3.3. Inhibition of Phage Infection Activity by Other Antibiotics

#### 3.3.1. Taketo and Watanabe, 1967, *Escherichia coli*, Nalidixic Acid

Taketo and Watanabe [83] reported that nalidixic acid at concentrations that were able to reduce *E. coli* CFUs after 60-min of exposure, and thereby presumably exceeded MIC (50 μg/mL), were also able to nearly or fully prevent the production of virions both by double-stranded phages (T2 and T5) and by single-stranded DNA phages (φR and φX174), but did not prevent the production of virions by RNA phages (β or 10). In these experiments, antibiotic was added 7 min after phages, with bacteria lysed by chloroform 60 min after antibiotic addition and prior to plating. The inhibition of phage T2 was, however, reversible given suspension of phage-infected bacteria into antibiotic-free media. Therefore, nalidixic acid appears to only reversibly inhibit phage bactericidal and indeed virion production ability, at least after 1-hour incubations with the antibiotic. Nalidixic acid, like ciprofloxacin, is an inhibitor of DNA gyrase and topoisomerase. Therefore, these experiments suggest a substantial impact of inhibition of DNA gyrase or topoisomerase on phage replication, though with caveats as well as corroborations as presented by Baird et al. [84], discussed as follows.

#### 3.3.2. Baird et al., 1972, *Escherichia coli*, Nalidixic Acid

Baird et al. [84] adsorbed phages (MOI < 1) for 5 to 7 min before exposing them also to 50 μg/mL nalidixic acid. They observed nearly consistent results with those of Taketo and Watanabe [83] for phage φR and perhaps for phage T5 as well. They also observed complete inhibition of *E. coli* phage T7 also using 50 μg/mL nalidixic acid and over 95% burst-size reduction with 10 μg/mL. The Baird et al. results for phage T2, however, seem to contradict those of Taketo and Watanabe, with burst size 34% of that without antibiotic. A possible explanation for this discrepancy is that of a need for prior equilibration of the antibiotic with bacterial cells, where a 30-min pre-incubation reduced burst sizes of phage T4—which was impacted identically to that of phage T2 given no pre-incubation—to only 2% of the burst without antibiotic treatment.

Inconsistently, no such pre-incubation was employed by Taketo and Watanabe. Baird et al. suggest, however, that adsorption by newly released virions—following lysis but prior to chloroform treatment at 60 min—could explain the loss of phage viability in the Taketo and Watanabe experiments as those authors did not dilute their infective centers in the course of their experiments, with naturally released virions therefore likely to have infected new bacteria, and thus may have been killed upon chloroform treatment. This hypothesis was tested and confirmed by Baird et al. In a separate experiment, the burst size of phage λ following induction was 2.5% that of the no-antibiotic control given dilution into 50 μg/mL nalidixic acid 7 min following the start of induction. This result suggests possibly a complete blockage of virion production given sufficient antibiotic pre-incubation. Although the Baird et al. results are not fully consistent with those of Taketo and Watanabe, they nevertheless are suggestive of a blocking, perhaps in full given sufficient pre-incubation with antibiotic, of virion production by at least some phages by the presence of 50 μg/mL nalidixic acid, though MIC was not determined.

#### 3.3.3. Valério et al., 2017, *Escherichia coli*, Various Antibiotics

Valério et al. [85] observed an absence of either phage-mediated killing of *E. coli* or phage ECA2 (10^7^ PFU/mL) population growth while in the presence of chloramphenicol or tetracycline at 1× MIC. They also observed an absence of phage population growth and probably a lack of phage-mediated bacteria killing in the presence of 1× MIC ciprofloxacin. Although these experiments were performed in phosphate-buffered saline, nevertheless phage population growth was substantial (1 log) and bacterial population growth occurred as well (up to 4 log), both in the absence of antibiotic presence. Therefore, the Valério et al. results are consistent with antibiotic interfering with phage infection activity, but possibly some of the observed antibiotic interference with phage infection activity was intensified given the low nutrient conditions that presumably were present during experiments.

#### 3.3.4. Summary of Studies of Inhibition of Phage Infection Activity by Other Antibiotics

The primary take-home message from this subsection are those of Baird et al. [84], finding that nalidixic acid can reduce the burst sizes of dsDNA phages, particularly given sufficient equilibration of antibiotic with bacteria prior to addition of phages. Taketo and Watanabe [83] failed to observe a similar inhibition of RNA phages by nalidixic acid.

### 3.4. Antibiotic Interference with Phage Pharmacodynamics, Conclusions

**Figure 1:**
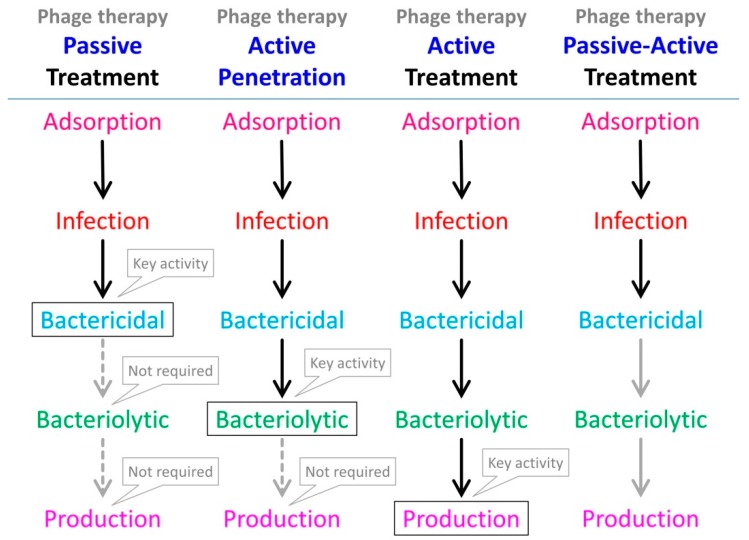
Progression of phage life cycles (top to bottom) and illustration of different phage therapy strategies (left to right) as requiring different degrees of phage infection activity. All terms refer to phage activities rather than antibiotic activities, including phage killing of bacteria (bactericidal), phage lysing of bacteria (bacteriolytic), and phage-infection generation and release of new virion particles (production). Boxes indicate key phage infection activities for a given strategy. Arrows indicate phage infection progressions from top to bottom, i.e., from bacterial adsorption, to bacterial infection, to bacteria killing, to lysing of bacteria, and then to release of new virions (production). Graying of arrows indicates a lack of requirement for this progression (dashed gray arrows) or lack of strict requirement (solid gray arrows) for a given phage therapy strategy (which are named across the top row). Passive treatment (first column) uses bacteria-overwhelming (inundative) phage dosing and thus requires only *bactericidal* activity from phage infections rather than necessarily also bacterial lysis or virion production. Active treatment (third column) does not use bacteria-overwhelming phage dosing so does require in situ phage virion *production*, often in substantial amounts. Active penetration (second column, but not otherwise discussed in the main text) is hypothesized as being involved in phage anti-biofilm activity and as being aided by phage *bacteriolytic* activity; it does not necessarily also require phage virion production. (Mixed) Passive-active treatment (fourth column, and also not otherwise discussed in the main text) is passive treatment that is hypothesized to be aided in its antibacterial activity by increases in phage numbers due to in situ phage virion production (solid gray arrows), which is unlike strictly passive treatments, which by definition do not necessarily involve such activities [94]. See Abedon and Thomas-Abedon [95] and Abedon [96,97] for review and further discussion. This figure is a modification of that presented in Abedon [97].

Given that phage bactericidal activity in the presence of antibiotics—antibiotics to which targeted bacteria are susceptible—can be difficult assess (Section 2), it is not surprising that there is a tendency among studies to focus instead on antibiotic-mediated diminishment of virion production. It is important to recognize, however, that at a minimum phage therapy efficacy requires only bactericidal activity (for so-called passive treatments) rather than necessarily virion production as well (for so-called active treatments) [92,93,94,95,96] (see Figure 1). Therefore, even evidence of dramatic reductions in phage virion production in the presence of antibiotics does not imply also a phage inability to contribute to the treatment of antibiotic-susceptible bacteria, also in the presence of antibiotic. Furthermore, we can speculate that under more complex treatment scenarios, such as of biofilms as considered in the following section, the bacteria most impacted by phages may be those that simply happen to be least impacted by antibiotics (Section 2). Therefore, we can question to what degree the findings reviewed in this section—that antibiotics can substantially interfere with phage infection activities—are highly relevant to phage therapy efficacy, especially phage therapy as involving application of relatively high phage doses (thereby allowing passive treatment), and particularly so given the lack of associated MIC determinations in many studies.

At the same time, these experiments, as listed in Table 1 and reviewed in this section, point to a utility, towards at least better appreciating potential in situ phage therapy pharmacodynamics, to assessing phage-antibiotic compatibility relatively early during the development of co-treatment protocols. This assessment can be accomplished, for example, by exploring single-step growth-type characteristics [90,91] in the presence of 1× MIC or greater antibiotic concentrations. 

## 4. Evidence of Phage-Infection Compatibility with Antibiotic Treatment

It is perhaps a naïve assumption that—at 1× MIC for a given antibiotic—bacterial physiology will be sufficiently negatively affected that support of phage infection activity will always be fully curtailed. MIC determinations, that is, generally involve macroscopic evidence of bacterial growth after a defined length of incubation, i.e., the formation of turbid broth cultures or colonies on agar after some period of incubation [46]. This blocking of macroscopically visible culture growth, however, does not necessarily mean that the affected bacteria are completely inhibited in their metabolism at 1× MIC, nor that phage infection activity therefore should be completely curtailed. Furthermore, MICs are determined under specific experimental conditions that are not necessarily replicated when phage infections are being tested for activity in an antibiotic’s presence. Less bacterial metabolic activity also may be required for a phage infection to be bactericidal than for it be bacteriolytic, and likely less metabolic activity to be bacteriolytic than to be virion productive. Therefore, e.g., a bacterial culture that is unable to replicate due to antibiotic presence, particularly antibiotic presence at approximately 1× MIC, might still be able to support, for example, phage-mediated bacteria killing, or even bacterial lysis or phage virion production. This section considers published evidence that one or more-fold MIC of antibiotic can still be compatible with phage infection activity, at least for some antibiotics under some conditions, though often with qualifications including as listed in this paragraph. Considered in this section are broth-culture, biofilm, and in vivo explorations of antibiotic impact on phage infection activities. See Table 2 for summary of the studies presented.

### 4.1. Broth-Culture Experiments

Broth-culture experiments have a utility of relative ease of execution, ready phage access to all bacteria present (assuming sufficient broth mixing), and relatively low physiological heterogeneity among those bacteria present. Ideally, experiments will explore the impact of measured 1× MIC or greater antibiotic concentrations on phage infection activity, with reporting of evidence for or against bactericidal, bacteriolytic, or phage virion production activities. Furthermore, characterizations of phage infection activities, at a minimum, should consist of phage single-step growth-like experiments [90,91], i.e., in which phage latent periods and burst sizes are determined, or instead other means by which phage bactericidal or bacteriolytic activities are directly measured. Here, with those issues in mind, I consider published broth-culture experiments that are suggestive of retention of phage pharmacodynamic impacts on bacteria in broth culture given antibiotic presence of 1× MICs or greater.

#### 4.1.1. Gage and Fujita, 1969, *Bacillus subtilis*, Nalidixic Acid

Perhaps equivalent to observations of rifampin-resistant phages (Section 3.2), Gage and Fujita [98] found that *B. subtilis* phage SPO1 (MOI = 6) was able to display bacteriolysis and virion production in the presence of nalidixic acid (50 μg/mL), “at concentrations that drastically inhibited *B. subtilis* DNA synthesis”. Latent periods, however, were approximately twice as long as without antibiotic, and burst sizes were reduced by roughly 30%. Consistent with display of this level of infection activity, the rates of DNA synthesis by antibiotic-treated, phage SPO1-infected bacteria were substantially higher than as seen with nalidixic acid treated, phage-uninfected bacteria: 0% vs 75% reductions at 5 μg/mL, respectively, and 30% vs 95% at 50 μg/mL. Nonetheless, and unfortunately, it is not possible from the data presented to conclude that antibiotic concentrations exceeded 1× MIC for the bacterial host.

#### 4.1.2. Baird et al., 1972, *Bacillus subtilis*, Nalidixic Acid

Baird et al. [84] found, similarly to Gage and Fujita [98], that *B. subtilis* phages φ29, SP50, and SP82 displayed burst sizes of 75%, 44%, and 46% that of the no-nalidixic acid controls as well as lysis delays in the presence of 50 μg/mL nalidixic acid. It is possible, though, that this relatively insubstantial reduction in phage virion production in the presence of nalidixic acid is a consequence of delays in equilibration of antibiotic with bacteria, thereby representing less antibiotic impact on phage virion production than might otherwise occur at that concentration. Specifically, Baird et al. also found that phage T4’s burst size infecting *E. coli* could be reduced from 34% to 2% of no antibiotic treatment given sooner exposure to antibiotic: delays of 5- to 7-min in antibiotic addition relative to phage addition (phages added first) versus 30 min delays in phage addition relative to antibiotic addition (antibiotic added first), respectively.

Baird et al. [84] also observed virion production activity by several additional phages also in the presence of nalidixic acid (φR, λ, T4, T5, and T7), though burst sizes tended to be somewhat reduced, with lysis delayed by 30 to 100 min. These latter observations come with the same concern as with phage T2—that antibiotic might not have been sufficiently equilibrated with bacteria prior to the start of phage infections to give rise to a full impact on phage infection activity. MICs were not reported.

#### 4.1.3. Pedrini et al., 1972, *Bacillus subtilis*, Nalidixic Acid

Pedrini et al. [99] considered the impact of nalidixic acid on the infection of phage SPP1 of *B. subtilis*. The antibiotic concentrations used was able to “block completely the DNA replication” of that host. As tested, this included 10 μg/mL of the drug, though at least a 10-min incubation was required before DNA replication ceased. An MOI of 1.5 was used and lysis was followed by optical density (OD_540_). While drops in OD, presumably as phage-induced, were fairly gradual, continuing for over at least a 100-min period, nevertheless with 10 μg/mL nalidixic acid added 10-min prior to phage addition, lysis kinetics were identical to as seen without the antibiotic present, and in the presence of 50 μg/mL nalidixic acid lysis was simply delayed by about 25 min rather than altered in the rate of lysis once begun. Phage SPP1 thus likely remains at least bacteriolytic even given exposure to nalidixic concentrations that are substantially more than that necessary to interfere with bacterial DNA replication. MIC for the host, though, was not reported.

#### 4.1.4. Price and Fogt, 1973, *Bacillus subtilis*, Nalidixic Acid

Price and Fogt [100] observed a reduction in the burst size of *B. subtilis* phage PBS2 (MOI = 5) of approximately 90% in the presence of simultaneously added 50 μg/mL nalidixic acid, but with burst size seemingly further reduced given antibiotic addition 5- or 15-min prior to phage addition. This is suggestive of substantial blocking of phage virion production but also (again) that delays may exist in equilibration of the antibiotic into cells following its addition to cultures. At this concentration of drug, bacterial DNA replication seems to have been blocked in uninfected cells. The same concentration, however, delayed but did not block phage-induced culture lysis, indicating a retention of at least bacteriolytic activity and presumably bactericidal activity as well (and hence the inclusion of this result under evidence of phage-antibiotic compatibility). Burst size, however, appeared to be substantially reduced.

This observation of presumptive bactericidal and/or bacteriolytic activities makes sense to the extent that phage gene expression, and thereby production of lysis-inducing proteins, is able to persist even if phage DNA replication is blocked. Addition of 20 μg/mL of nalidixic acid, described as “low levels”, by contrast blocked bacterial DNA replication in uninfected bacteria for over 70 min, but did not block bacterial DNA replication for over 90 min. This lower antibiotic concentration was associated with burst size reductions of about 50% given simultaneous phage and antibiotic addition, or greater reductions (~70%) when phages were added 5- or 15-min after antibiotic, though there was no associated delay in lysis. It is uncertain to what degree antibiotic concentrations of 50 μg/mL may have exceeded MIC. These results nevertheless are consistent with retention of at least some phage antibacterial activity despite nalidixic acid presence, and to a degree even retention of virion production by this phage in the presence of 50 μg/mL of this antibiotic.

#### 4.1.5. David et al., 1980, *Mycobacterium aurum*, Various Antibiotics

David et al. [101] found that the concentration of isoniazid required to reduce phage burst sizes by 50% was over two orders of magnitude greater than the MIC for this drug (*Mycobacterium aurum* as host bacterium and infection by phage D29). This is consistent with cell-wall synthesis inhibitors requiring a fair amount of time before they affect bacterial functioning. For the antibiotic ethambutol, the equivalent ratio to MIC was so high that it was not determinable. This drug too is a cell-wall synthesis inhibitor. Dapsone gave about an order of magnitude greater phage inhibitory value (50% reduction in burst size) than the measured MIC. Although dapsone is not a cell-wall synthesis inhibitor, it is an enzyme inhibitor.

For all other antibiotics tested (clofazimine, colistin, rifampin, and streptomycin), ratios were closer to 1, suggesting indeed an inhibition of at least phage virion production at antibiotic concentrations close to MIC. Of these, colistin and streptomycin had the greatest impacts on phage virion production versus bacterial replication, with the ratio to MIC being 0.78 and 0.54, respectively, e.g., as versus 166 for isoniazid. The colistin result is potentially odd, however, given that this antibiotic too acts on the bacterial cell envelope rather than interfering internally, though then again mycobacteria do not have Gram negative cell envelopes, where Gram-negative pathogens and their outer membranes are the normal targets of colistin. These results are clearly indicative nevertheless of a potential—while infecting this bacterium—for this phage to replicate in the presence of certain antibiotics (dapsone, ethambutol, and isoniazid). A caveat, however, is that drugs were not added until after phage adsorption was considered to have occurred, and thus antibiotic was delayed in its addition, potentially resulting in greater phage infection activity than otherwise might have been seen versus, e.g., given pre-incubation of antibiotic.

#### 4.1.6. Lavysh et al., 2017, *Bacillus subtilis*, Rifampin

Lavysh et al. [102] studied a *B. subtilis* phage, AR9, which displayed incomplete inhibition of phage virion production given infection exposure to rifampin (50 μg/mL). Indeed, in single-step growth experiments an approximately ten-fold reduction in burst size was reported, as representing a total of only about eight virions produced per phage-infected bacterium, though it is not obvious when antibiotic was applied relative to phages. Antibiotic concentrations at 50 μg/mL alone resulted, after 10-min exposure, also in ~100-fold reductions in cell viability. Therefore, the authors suggest that phage AR9 may in fact not be rifampin susceptible, e.g., as potentially due to the phage virion carrying its own RNA polymerase, but with the decrease in phage burst size related instead to cell killing by the antibiotic. This hypothesis would seem to be somewhat consistent with the indicated 1-log drop in pre-burst phage infective centers with rifampin treatment (their Figure S2), and this explanation would be rather than due to more specific inhibition by rifampin of phage functioning during infection.

Inconsistent with the infection death hypothesis, using a ~ten-fold less rifampin-susceptible host, Lavysh et al. [102] still see a roughly ten-fold drop in burst size with rifampin treatment, but without the pre-burst reduction in infective centers (again, their Figure S2). Interpretation of these results in terms of retention by phage AR9 of infection activity in the presence of rifampin therefore would appear to be complicated. Either what is being observed is reduced virion production in the presence of substantial amounts of rifampin, by a rifampin-susceptible phage, or instead a more general failure of host physiology affecting the burst size of an otherwise rifampin-resistant phage. Still, some phage virion production is observed in the presence of rifampin, though MICs for the hosts were not explicitly determined.

#### 4.1.7. Matsui et al., 2017, *Ralstonia solanacearum*, Rifampin

Matsui et al. [68] tested the ability of phages ΦRP12 and ΦRP31 of *R. solanacearum* to replicate in the presence of up to 20 μg/mL of rifampin (MIC = 5 μg/mL). At 1× or 2× MIC, resulting titers were reduced by about one log, whereas at 4× MIC, i.e., 20 μg/mL, they were reduced by roughly 2 logs. The authors suggest that these results are an indication that these phages, like phage PBS2 of *B. subtilis* [89] or phage φKZ of *P. aeruginosa* [74], carry their own RNA polymerases, particularly as other phages they tested, ΦRSB1, ΦRSL1, ΦRSL2, and ΦRPSF1, were completely blocked in terms of phage virion production at 1× MIC. It is notable, however, that phage virion production for phages φKZ and PBS2 was demonstrated at much higher rifampin concentrations, i.e., 100 and 400 μg/mL, respectively, with relatively little decline in virion production, though unfortunately in neither of these latter cases was MIC reported. As no RNA polymerase was demonstrated for the *R. solanacearum* phages—unlike for phage φKZ, for which candidate subunits were identified and for which no bacterial promoters could be identified—it is more difficult to conclude that phages ΦRP12 and ΦRP31 truly carry their own enzyme within virions or instead for some reason are simply in some manner less susceptible to this antibiotic than the other phages tested.

#### 4.1.8. Oechslin et al., 2017, *Pseudomonas aeruginosa*, Ciprofloxacin and Meropenem

Oechslin et al. [103] treated broth cultures of *P. aeruginosa* with combinations of a phage cocktail (10^8^ PFU/mL) and either ciprofloxacin or meropenem (both at 2.5× MIC), presumably with each added simultaneously. After six hours of exposure, bacterial numbers were reduced by roughly 6 logs in both cases following either phage-only treatment or combined treatment, versus less than 1-log reductions by antibiotic alone, and approximately 1-log increases without treatment. As the calculated MOI was only 1, the 6-log declines in CFUs are indicative of phage virion production during treatments, both with and without antibiotic presence.

#### 4.1.9. Jansen et al., 2018, *Acinetobacter baumannii*, Ciprofloxacin, Colistin, and Meropenem

Jansen et al. [104] followed broth cultures of *A. baumannii* for 16 h in the presence of phage KARL-1 added at various starting MOIs (all less than 1, the highest being 0.1) and various concentrations of the antibiotics ciprofloxacin, colistin, or meropenem (ranging from less than to more than MIC; the bacterial isolate used is described as multi-drug resistant, but nevertheless antibiotic concentrations used in experiments exceeded MICs, so this study is included here). With antibiotics alone there was substantial resulting culture turbidity at all treatment concentrations, though especially with ciprofloxacin there was some increasing reduction in turbidity with increasing antibiotic concentration. With phage-antibiotic combinations there generally was less turbidity at the end of experiments than with antibiotic alone, even starting with higher antibiotic concentrations (i.e., >MIC) or lower phage MOIs (down to 10^−7^). Even if differences in end-point turbidities were a consequence of increased likelihoods of mutation to resistance without combination therapy versus with, these results are still suggestive of phage population growth in the presence of antibiotic since phage concentrations would need to exceed MOI = 1 for these combination treatment effects to be observed. What is uncertain, however, is whether this proposed phage population growth occurred in association with antibiotic-susceptible versus antibiotic-resistant bacteria. Their Figure S5, however, is at least suggestive that this latter hypothesis is not correct. Instead, it would appear that experimental conditions might have differed from those that were used to determine MICs such that antibiotic-susceptible bacteria nevertheless were able to at least increase in turbidity over time in the presence of all antibiotic concentrations tested, though mostly with less of an increase than as observed with the untreated control culture (colistin-treated cultures were an exception to this latter point, where increases in culture turbidity without phage treatment, with all but the highest colistin concentrations similar to the untreated control). The lower impact, i.e., higher resulting culture turbidities that were observed with the lowest starting phage MOIs (in combination with treatment with ciprofloxacin or colistin), seems to be due to delays in phage population growth to MOIs of greater than 1, that is, as associated with lower phage starting numbers, thereby resulting in less efficient culture clearing.

#### 4.1.10. Lopes et al., 2018, *Escherichia coli*, Ciprofloxacin

Lopes et al. [105] found evidence of an over 1 log but nonetheless only transient (not long lived) increase in phage ELY-1 numbers in the presence of both 1× MIC ciprofloxacin and *E. coli*. In the same experiments, phage impact on the *E. coli* target in combination with ciprofloxacin was greater, by nearly 1 log, than ciprofloxacin impact alone, though phage-antibiotic combination treatments reduced bacterial numbers somewhat less than phage impact alone. As starting phage titers were only about 10^6^ PFU/mL, these results, in combination, are consistent with the occurrence of some phage virion production in the presence of ciprofloxacin, at least at 1× MIC.

No increase in phage numbers was seen with 2× MIC ciprofloxacin, and this was versus an approximately 3-log increase in phage numbers without ciprofloxacin present. No difference in terms of impact on bacterial presence was observed with ciprofloxacin alone versus with phages present as well, also given antibiotic concentrations of 2× MIC. Therefore, overall, at least a small amount of phage virion production appears to be present at 1× MIC ciprofloxacin in this system, but not at 2× MIC.

#### 4.1.11. Summary of Evidence of Phage Infection Activity in Broth in the Presence of Antibiotics

The results presented in this subsection are consistent with at least some phage infection activity in broth bacterial cultures, and specifically phage virion production at antibiotic concentrations of at least 1× MIC. Evidence is also provided that positive phage infection-activity results can be reduced given longer pre-incubations of antibiotic with bacteria prior to phage application. Furthermore, while bacteriolytic activity can be retained in the presence of antibiotic, burst sizes also can be reduced and latent periods extended. Different phages also appear to display different levels of sensitivity to antibiotic presence. In my opinion, the primary take-home messages of this subsection are that antibiotics at concentrations that inhibit bacterial replication do not necessarily also fully interfere with phage infection activity, including in terms of virion production, but that it is important to confirm that this result is retained even given delays between antibiotic addition and subsequent phage addition.

### 4.2. Biofilm Experiments

A number of studies have been recently published that employ combinations of phages and antibiotics to treat in vitro-grown bacterial biofilms. Interpretation of results of these experiments, in terms of phage-antibiotic interactions and resulting pharmacodynamics, can however be challenging due to the possible heterogeneity of these cultures in terms of the susceptibility of individual bacteria to phages, antibiotic, or both. In addition is the issue of the potential for phages to carry biofilm-matrix degrading enzymes including in association with virions (Section 2). Biofilms also can be notoriously tolerant of even high densities of antibiotic, e.g., [52,54,111], where antibiotic impact in any case can be highly dependent on the conditions a bacterium is experiencing [46,59]. Various phage impacts on the presence of biofilm bacteria are discussed in this subsection in terms of possible retention of phage infection activity despite antibiotic co-treatments. 

#### 4.2.1. Bedi et al., 2009, *Klebsiella pneumoniae*, Amoxicillin

Bedi et al. [106] found somewhat greater phage impact in the presence of 2× MIC amoxicillin on *K. pneumoniae* one-day-old biofilms—in terms of reductions in numbers of CFUs—than with either phage or antibiotic treatment alone (the name of the phage used does not appear to have been specified by the authors). This result is suggestive of retention of phage anti-biofilm activity despite antibiotic presence. Given that phage doses were of only MOI = 0.01, this result is consistent also with phage virion production in association with the treated biofilm. Note however the potential for heterogeneity in bacterial physiology or in antibiotic susceptibility within biofilms, or indeed the potential for antibiotic to decay over time, which makes it difficult to tell whether the individual bacteria supporting such phage virion production were also bacteria that at the same time were highly affected by antibiotic. Furthermore, amoxicillin as a cell-wall synthesis-inhibiting antibiotic potentially allows phage infection until bacteria either have been killed or nearly killed by amoxicillin activity.

A concern with experiments in which treatments take place overnight or longer is the potential for regrowth of biofilm by resistant bacteria (Section 2). That is, given a combination of phage and antibiotic application, the likelihood of mutation to resistance to both agents typically would be lower than the likelihood of bacterial mutation to resistance to each agent individually (e.g., 10^−5^ mutations per cell division versus 10^−10^). As a result, differences in numbers of CFUs between antibiotic-only and phage-plus-antibiotic treatments, particularly for experiments done within relatively small volumes, could be due solely to regrowth of antibiotic-resistant bacteria (e.g., 10^−5^ mutations per cell division) but no regrowth of bacteria that are both phage and antibiotic resistant (e.g., 10^−10^ double mutations per cell division). In other words, differences in numbers of CFUs at the point of bacterial enumeration could be due to one treatment having a greater potential for biofilm elimination to be reversed over time (because of growth of resistant bacteria), and this is rather than due to differences in more immediate pharmacodynamic antibacterial impacts.

Phage population growth and therefore virion production nevertheless still must have occurred in the Bedi et al. [106] experiments given the low initial phage MOI used, that is, for phage numbers to have become sufficiently high to interfere with bacterial resistance evolution in the course of the phage-plus-antibiotic treatment. Therefore, even given the potential for regrowth of bacterial resistance mutants, these results should still be consistent with phage virion production in the presence of what at least initially was 2× MIC amoxicillin. Again, however, it remains uncertain to what degree the individual bacteria supporting this presumed phage virion production were fully affected by the antibiotic treatment, e.g., such as due to antibiotic decay over time.

#### 4.2.2. Verma et al., 2009, *Klebsiella pneumoniae*, Ciprofloxacin

Verma et al. [107] treated 12-hour *K. pneumoniae* biofilms with a combination of phage KPO1K2 (MOI = 1) and ciprofloxacin (10 μg/mL; MIC for planktonic bacteria = 0.625 μg/mL). Ciprofloxacin after 0.5 to 3 h of treatment reduced biofilm CFUs by roughly 1 log. This reduction, however, is relative to the untreated control rather than to bacterial densities that were present at the start of treatments. Ciprofloxacin at this density thus likely was preventing biofilm growth, but otherwise may not have actually reduced bacterial numbers. Reductions associated with phage treatment were roughly 3 to 4 log relative to the bacterial concentrations at the start of treatment, and these amounts were regardless of whether antibiotic was present. Such levels of reduction would appear to be consistent not only with display by this phage of bactericidal activity in the presence of antibiotic, but with phage virion production as well, i.e., as an MOI of 1 alone is insufficient to result in multi-log reductions in bacterial densities.

An alternative hypothesis is that phage presence resulted in reduced resistance by biofilm to removal during washing, with washing preceding enumeration of biofilm bacteria. In particular, phage KPO1K2 was found to give rise to a substantial halo surrounding plaques [120], as is suggestive of phage encoding of biofilm-matrix degrading enzyme. These results, particularly absent explicit documentation of phage population growth in association with ciprofloxacin, thus should be viewed as inconclusive in terms of assessing either phage bactericidal or virion production abilities in the presence of antibiotic.

#### 4.2.3. Rahman et al., 2011, *Staphylococcus aureus*, Azithromycin, Rifampin, and Vancomycin

Rahman et al. [108], using 10× MIC rifampin against 24-h *S. aureus* biofilm, observed approximately 1-log greater biofilm reduction in terms of CFUs with phage SAP-26 presence than without. This difference was seen after 8 h of treatment, but not after 4 h, and continued through 12 and 24 h. As the phage titer appeared to be in excess of 10^8^ PFU/mL (and I speculate was in fact 10^9^ PFU/mL as 10^8^ PFU seem to have been suspended in 100 μL), these results are indicative of phage anti-biofilm activity, and possibly phage bactericidal activity, but not necessarily also phage bacteriolytic or virion production activity.

Greater killing with phages being present than without phages being present, in both cases in the presence of rifampin, was also seen after 24 h of treatment as determined via a 2, 3, 5-triphenyltetrazolium chloride-based assay. Here, however, differences were only about two-fold, i.e., ~72%, ~60%, and ~35% cells living given phage-only, rifampin-only, or phage-plus-rifampin treatments. For azithromycin, these numbers instead are ~72%, ~75%, and ~40%, also suggesting phage activity compatibility with the presence of antibiotic. With vancomycin the numbers instead are ~72%, ~83%, and ~60%, which is less convincing of such compatibility of phage infection activity with antibiotic presence.

#### 4.2.4. Coulter et al., 2014, *Escherichia coli* and *Pseudomonas aeruginosa*, Tobramycin

Coulter et al. [39] found that tobramycin (2 μg/mL) was able to reduce 48-h *E. coli* biofilm bacterial numbers by about 2.5 log relative to an untreated control. In the presence of both phage T4 and the antibiotic, however, an approximately 6.5-log reduction in biofilm bacteria numbers was observed. This result should be indicative of phage population growth since the starting phage MOI was 0.01, which on its own at best should result in a killing of about 1% of the bacteria present (Section 2). An equivalent experiment in the same study with *P. aeruginosa* and phage PB-1 (and 0.5 μg/mL tobramycin), by contrast, yielded somewhat weaker evidence of phage activity in the presence of tobramycin. 

#### 4.2.5. Kaur et al., 2014, *Staphylococcus aureus*, Linezolid

Kaur et al. [109] is a prevention of bacterial surface adherence study (*S. aureus*). Phage MR-5 and linezolid were applied to orthopedic-grade stainless steel Kirschner wires, with colony counts of adhered bacteria made after 6-, 24-, and 48-hs of incubation in the presence of bacteria. Both phage and antibiotic were applied to wires within hydroxypropyl methylcellulose hydrogels. Linezolid alone (5% *w*/*w*) and phages alone (1.5 × 10^9^ PFU/mL) allowed similar levels of bacterial colonization of wires. A combination of the two agents, however, resulted in about 1-log less colonization (all statistically significantly different from colonization of untreated wires). Bacteria which failed to encounter or be affected by linezolid upon attempting to adhere to wire surfaces thus seemingly could instead have encountered a phage, resulting in the additional reduction in CFUs.

The implication of this result is retention of phage bactericidal activity in the presence of the antibiotic. Presumably, however, for phages to contribute to reducing bacterial colonization of wires, then it would have been those bacteria which were least impacted by linezolid that would have been most affected by phages, else those bacteria should have been sufficiently impacted by linezolid that phage infection would not have resulted in excess bacterial losses (that is, a phage cannot be solely responsible for preventing colonization by an individual bacterium if that colonization also has been prevented by linezolid). That interpretation would call into question whether phage activity truly was retained given individual bacteria exposure to at least an MIC of antibiotic, with antibiotic concentration otherwise reported as “much higher than the MIC level”. It is also conceivable that phages were able to better gain access to bacteria and/or for subsequent bacteria killing to have occurred in the course of bacterial enumeration [121], i.e., as following biofilm disruption, rather than within hydrogels, again calling into question whether phage bactericidal activity was retained in the presence of substantially antibiotic-affected bacteria. In any case, as wires and bacteria were suspended in phosphate-buffered saline during experiments, presumably phage infection activity, and also antibiotic activity, may not have been robust, regardless, until the enumeration step.

#### 4.2.6. Singla et al., 2016, *Klebsiella pneumoniae*, Amikacin

Singla et al. [110] treated *K. pneumoniae* biofilm grown on microtiter plates with a combination of phage KPO1K2 (MOI = 1 relative to bacterial density found in untreated biofilm) and amikacin (40 μg/mL). Phages were either free or liposome entrapped upon application to biofilms, and biofilms were grown between one and seven days prior to the start treatment. The impact of antibiotic generally was less than that of phages, though not necessarily statistically significantly less, and free phages may have been slightly less effective as anti-biofilm agents than liposome-entrapped phages, depending on the age of the biofilm. That antibiotic treatment alone resulted in reductions in bacterial numbers found in biofilms, however, is suggestive that the dose of amikacin employed exceeded MIC, though MIC was not otherwise determined. Treated biofilms generally had bacterial counts that were closer to those of the negative-treatment controls, the older the treated biofilm, ranging from over 3-log reductions for one-day-old biofilms to roughly 1-log reductions for seven-day-old biofilms (the latter not statistically significant especially in terms of free phage or antibiotic impact).

Experiments by Singla et al. [110] consisting of phage treatment in combination with antibiotic were done without phage-only or antibiotic-only controls. In comparing these experiments with separate individual-treatment experiments (i.e., as discussed in the previous paragraph), it would appear that there was little difference between the impact of free phages and antibiotic added in combination versus acting alone. When treatment phages were entrapped in liposomes, however, then the combination of phages and antibiotic resulted in bacterial counts which were reduced by an additional 1 log or more. This latter result is at least suggestive of phage bactericidal activity. Given that an MOI of 1 should result in at best only 63% of the bacteria initially becoming phage infected (a 0.43 log reduction; Section 2), this result is suggestive also that phage virion production in the presence of antibiotic may have occurred, though the occurrence of such growth was not explicitly determined.

Why this possible phage virion production should have occurred in the presence of antibiotic especially in the case where phages were liposome entrapped, but not without liposome entrapment, is uncertain. This is especially uncertain given that, when phages were employed without antibiotic present, reductions in bacterial numbers ranged, as noted, from 1- to 3-log (differences between results of treatment with and without liposome entrapment in the presence of amikacin was not determined in terms of statistical significance). Therefore, these experiments appear to be consistent with phage bactericidal and possibly also virion production activity in the presence of amikacin (though perhaps less virion production than as seen without antibiotic), but the result is complicated by its dependence on phage entrapment within liposomes. I speculate that the improved pharmacodynamic impact with phage liposome entrapment in the presence of amikacin, however, is a consequence of better phage penetration to bacteria. That is, either for spatial reasons (as consistent with authors’ suggestion) or for temporal reasons—as in declines in active antibiotic over time in combination with delayed phage adsorption to bacteria when liposome entrapped—bacteria were less affected by antibiotic at the point of phage encounter given phage liposome entrapment. A further complication is that phage KPO1K2 may encode an anti-biofilm-matrix hydrolase [120] (see Section 4.2.2).

#### 4.2.7. Chaudhry et al., 2017, *Pseudomonas aeruginosa*, Various Antibiotics

Chaudhry et al. [18] determined the number of phages present after 48 h of treatment following their exposure to 48-h *P. aeruginosa* biofilm in the presence of various antibiotics, with phages NP1 and NP3 applied in combination to 10^6^ PFU/mL. Specifically, they found that while ciprofloxacin and tobramycin blocked phage production at 8× MIC for these antibiotics, that blockage of phage production was not also seen at 1× MIC. Furthermore, for ceftazidime and colistin, phage production was seen even at 8× MIC, though with gentamycin no phage production was seen even at 1× MIC. This evidence, however, is not fully supportive of the idea that phage infections necessarily will be active in the presence of these antibiotics even at 1× MIC, given the potential for biofilm heterogeneity and antibiotic tolerance (Section 2). In addition, phage numbers were determined after 48 h of incubation, at which point phage-sensitive but antibiotic-resistant bacteria may have arisen, or instead antibiotic over the course of assays may have decayed. Either of these cases could potentially support phage population growth in association with individual bacteria which are less antibiotic affected (in addition, if phages are unable to replicate and are otherwise present starting at low titers, then antibiotic resistance need not evolve at the same time as phage resistance, that is, for some degree of biofilm regrowth to occur in the presence of both agents). It is possible, therefore, that what was observed in these experiments was something other than production of new phage virions during infection of highly antibiotic-impacted bacteria. 

Contrasting that conclusion, for ceftazidime and colistin 4-log increases in phage numbers were observed in the presence of biofilm and 8× MIC antibiotic concentrations, which suggests an involvement of all of the bacteria present in the observed phage population growth, i.e., as this was similar to the amount of phage population growth seen without antibiotic presence in a different experiment. This result therefore is highly suggestive of at least some phage population growth in association with fully antibiotic-exposed bacteria. As both antibiotics are externally rather than internally acting, however, this result may not be entirely unexpected, as consistent with the results of David et al. [101], as discussed above. That is, a substantial amount of phage virion production might have taken place prior to bacteria being highly affected by these two antibiotics (though note that David et al. did *not* actually see equivalently enhanced phage virion production given treatment with colistin). In addition, for all five antibiotics at 1× MIC concentrations, phages were able to increase in number roughly 2- to 3-log fold over a 12-hour period following exposure to eight-hour biofilms growing on a layer of epithelial cells.

Clearly phage antibacterial activity or even production of new phage particles does not appear to be blocked absolutely under all circumstances of antibiotic co-treatment, as tested in this study. Nevertheless, for at least some of the experiments presented, questions remain as to the degree that antibiotics were affecting those individual bacteria responsible for supporting phage virion production.

#### 4.2.8. Kumaran et al., 2018, *Staphylococcus aureus*, Vancomycin

Kumaran et al. [111] found that treatment of a 24-h *S. aureus* biofilm with a combination of vancomycin and phage SATA-8505 resulted in roughly a half-log greater reduction in numbers of bacteria versus vancomycin alone. This was seen at vancomycin concentrations of 32, 64, and 128 μg/mL, while less reduction was seen at 256, 512, and 1024 μg/ml (MIC for non-biofilm bacteria was 4 μg/mL). Given that dosing was with only 10^6^ PFU/ml, these results are suggestive of a retention of not just phage bactericidal activity but also phage virion production despite vancomycin presence. Similar results, however, were absent in the presence of equivalent quantities of cefazolin, dicloxacillin, linezolid, or tetracycline. As vancomycin is a cell-wall-targeting antibiotic, it is conceivable that suggested phage population growth occurred in these experiments prior to substantial vancomycin impact on bacterial metabolism.

#### 4.2.9. Yazdi et al., 2018, *Proteus mirabilis*, Ampicillin

Yazdi et al. [112] treated *P. mirabilis* biofilm with phage vB_PmiS-TH (MOI = 1 or MOI = 100) and ampicillin (1× or 15× MIC). With MOI = 100, either with or without 1× MIC ampicillin, where 1× MIC ampicillin alone had little impact on biofilm bacteria, there was about 90% removal of biofilm after eight hours, as determined via crystal violet staining, but only 70% removal after 24 h. By contrast, with MOI = 100 and 15× MIC, there was no difference between eight and 24 h given this combined treatment, with 90% removal in both cases. Therefore, with time, and without substantial inhibition of bacterial growth as accomplished via inclusion of 15× MIC ampicillin, there appeared to be grow back of what we can presume are phage-resistant bacteria, i.e., 90% biofilm removal by eight hours, then only 70% biofilm removal by 24 h with MOI = 100 and antibiotic concentrations of either 1× or 0× MIC (that is, from the publication, “MOI 100 + AMP 16 μg/mL” or “MOI 100”, respectively) versus MOI = 100 and antibiotic concentration of 15× MIC (“MOI 100 + AMP 246 μg/mL”) for which no grow back was observed. In the same experiment, with 15× MIC ampicillin, there was somewhat more biofilm removal after eight hours with MOI = 1 (50%) or MOI = 100 (90%) relative to ampicillin alone (20%). The latter results too are suggestive of phage bactericidal activity in the presence of antibiotic. 

#### 4.2.10. Chang et al., 2019, *Pseudomonas aeruginosa*, Ciprofloxacin

Chang et al. [113]—with 24-h treatment of 48-h *P. aeruginosa* biofilms—saw greater reductions in biofilm biomass or biofilm viability with phage treatment (a cocktail, with 10^8^ PFU/mL) in combination with 1× MIC ciprofloxacin versus phage-only treatments, the latter which generally had little impact. These results would seem to imply that phages or antibiotic are unable to remove bacteria alone, and presumably thereby phages were unable to display bactericidal or bacteriolytic activity except when the other was also present. An alternative explanation is that with combination therapy then resistant bacteria are unable to reestablish biofilms following biofilm removal due to a low potential for bacteria to mutate simultaneously to both phage and antibiotic resistance (Section 2). This explanation, however, is problematic given that treatment was with a phage cocktail which itself should, at least ideally, interfere with bacterial evolution of phage resistance, and that one-half MIC ciprofloxacin in combination with phages gave similar results to 1× MIC. Although overall it is difficult to infer what might be underlying the results observed in this study, explanations nevertheless would seem to support some sort of phage anti-biofilm activity in the presence of 1× MIC antibiotic. This activity does not necessarily include virion production, however, given the high numbers that phages were dosed with. Indeed, whether this activity was explicitly phage-infection related versus strictly enzymatic in this study is difficult to conclude. An important additional caveat is that these anti-biofilm experiments possibly were conducted in buffer rather than in broth.

#### 4.2.11. Dickey and Perrot, 2019, *Staphylococcus aureus*, Various Antibiotics

Dickey and Perrot [34] tested in total eight antibiotics at 2× and 10× MIC against *S. aureus* biofilm, in combination with a phage they described as “PYO”, which they isolated from a Republic of Georgia commercially available phage cocktail of the same name. These antibiotics are ciprofloxacin, daptomycin, erythromycin, gentamycin, linezolid, oxacillin, tetracycline, and vancomycin. Phage titers after 48 h (Table 3) can be compared to those present at the start of experiments (<10 ^7^ PFU/mL). The same caveats about what these increases in phage numbers mean are applicable to these results as to those presented by Chaudhry et al. [18], etc., though there appears again to be strong indication of a potential for phage virion production despite antibiotic presence. Indeed, as measured explicitly in the same experiments, phage infection activity for a majority of the antibiotics tested tended to not be reduced given antibiotic presence at 2× MIC. For daptomycin and oxacillin there also appeared to be phage virion production even at 10× MIC. Therefore, these experiments seem to support a potential for phages in association with biofilm to produce new virions in the presence of a variety of antibiotics, though as above it is uncertain to what degree the bacteria supporting phage population growth are individually affected by the antibiotic present. At the same time, it is clear that 10× MIC for certain antibiotics, notably erythromycin, gentamycin, and linezolid, had substantial antagonistic impacts on phage population growth.

#### 4.2.12. Issa et al., 2019, *Pseudomonas aeruginosa*, Ciprofloxacin

Issa et al. [114] applied various phages at a titer of 10^3^ PFU/mL along with 1× MIC ciprofloxacin (0.5 μg/mL) and characterized the ability of simultaneously supplied *P. aeruginosa* to form biofilms (as then measured after 24 h of treatment). Approximately half as much biofilm biomass was present for all five tested phages (ΦPT-18[b], ΦPT-20[a], ΦPT-1S[a], ΦPT-5[a] and ΦPT-2[b]) in the presence of ciprofloxacin in comparison with ciprofloxacin alone. No phage-only controls are presented with the shown experiment, but previous experiments indicated that phage-only treatment is less effective than ciprofloxacin treatment alone. Given the low numbers of phages provided, these results are suggestive that phage virion production indeed may have occurred despite ciprofloxacin’s presence. That the bacteria formed biofilms at all in the presence of ciprofloxacin, however, is puzzling, perhaps suggesting that it was instead ciprofloxacin-resistant mutants which formed the biofilm, and also ciprofloxacin-resistant mutants which supported the proposed phage population growth.

#### 4.2.13. Summary of Evidence of Phage Infection Activity in Biofilms in the Presence of Antibiotics

The results reviewed in this subsection are highly suggestive that phage infection activity can persist in association with bacterial biofilms despite presence of antibiotic concentrations of 1× MIC or greater. Nevertheless, issues exist concerning the heterogeneity of biofilms and cultures, both spatially and temporally. These are in terms of the degree that constituent bacteria are potentially unevenly impacted by antibiotic while supporting phage infection activity, as well as the potential for antibiotics to decay such as to subinhibitory levels over time. In either case, phage infection activity might be occurring in association with antibiotic not inhibitorily affecting bacterial hosts despite initial antibiotic concentrations at or exceeding 1× MIC. Additional issues are the presence of biofilm-matrix degrading enzymes in at least some phage formulations as may mimic phage bactericidal activity, and also the potential for antibiotic-resistant bacteria to sufficiently grow in number over time to support phage virion production in an antibiotic-unaffected manner. Ideally, therefore, determinations of antibiotic impact on phage infection activity would be corroborated at the very least with simplified, shorter term determinations of phage-antibiotic compatibility such as the above-mentioned single-step growth experiments [90,91].

### 4.3. Phage-Antibiotic In Vivo Compatibility

For phage therapy to be effective, then phages at a minimum need to reach targeted bacteria and then kill those bacteria. Generally, the killing process involves several relatively complex physiological steps that are highly dependent on various target-bacterium-supplied functions, especially as resulting in phage gene expression. In addition, to some degree phage in situ replication can be helpful or even crucial to phage therapy success, resulting in so-called active treatments [92,93,94,95,96]. Phage replication along with virion production, however, generally should be associated with more bacterial metabolic functioning than simply phage bactericidal activity. In this section, I consider animal models of phage therapy in which potentially clinically effective dosings of antibiotics are provided along with phages as co-therapies. Given the greater complexity of these models relative to in vitro studies (Section 4.1 and Section 4.2), it can be even more difficult to ascertain degrees of retention of phage infection activity in the presence of antibiotic. Nevertheless, as above, evidence of in situ phage replication, towards greater phage titers than are present immediately following phage application, can serve as evidence for at least some degree of phage infection activity and associated pharmacodynamic impact despite presumed concurrent antibiotic negative impacts on the metabolic functioning of targeted bacteria. The issue of whether antibiotics and phages are together robustly impacting the same individual bacteria, however, can be even more difficult to answer with animal models than with in vitro biofilm studies (the latter, Section 4.2).

#### 4.3.1. Chhibber et al., 2008, *Klebsiella pneumoniae*, amikacin

Chhibber et al. [115], using a mouse, *K. pneumoniae*, lobar-pneumonia model, found no difference in phage SS impact on bacteria with versus without antibiotic presence. A reported 1× MIC amikacin (3.75 mg/25 g) was supplied via intraperitoneal delivery at the same time as phages (10^9^ PFU), also intraperitoneally, both in association with a simultaneous intranasal bacterial challenge (5 × 10^6^ CFU). Bacterial counts on Day 5 with phage treatment were 5-logs, or more, lower relative to the no-treatment control. Clearly phage bactericidal activity therefore was retained in this model in association with antibiotic. Antibiotic alone, however, had little impact on bacterial numbers. Evidence also is provided that, in the absence of bacteria, phages could concentrate in lung tissue, up to nearly 10^12^ PFU/g. As a result, it is uncertain whether phage replication may have occurred in the presence of antibiotic to result in observed declines in bacterial presence versus phage concentration to inundative titers in the lungs by other means, or, if phage replication did occur, whether antibiotic concentrations in contact with phage-infected bacteria were still equal to at least 1× MIC at the time of that replication. In addition, measured reductions in bacterial loads relative to untreated controls—given phage-only or combined treatments—did not begin until Day 2 of treatment, and reductions were not substantial, relative to bacterial numbers as present on Day 1, until Day 3 of treatment. Over this time frame, antibiotic concentrations likely would have declined to below 1× MIC, as evidenced by bacterial growth in association with the amikacin-only treatment group by Day 2. Therefore, evidence that phage replication occurred in association with 1× MIC or greater concentrations of amikacin in this study at best is not robust.

#### 4.3.2. Chhibber et al., 2012, *Staphylococcus aureus*, Linezolid

Chhibber et al. [116] treated a *S. aureus* model diabetic foot infection, mouse model, using a combination of phage MR-10 (10^6^ phages locally applied but 10^8^ PFU/mL) and linezolid (25 mg/Kg, orally dosed), with treatment starting 30-min post bacterial challenge. Evidence of phage virion production in the presence of antibiotic at best is slight as determined by phage titers found locally, as too is evidence of phage replication in terms of reductions in CFUs, also as found locally, where reductions were not substantial relative to likely starting bacterial densities. Combination treatments reduced bacterial densities by about 1-log more than phage- or antibiotic-alone treatments, however. This latter result, particularly as it occurred within one day of the bacterial challenge, and at the start of treatments, is suggestive of phage bactericidal activity in the presence of both antibiotic and reasonably antibiotic-affected bacteria as found in vivo.

#### 4.3.3. Yilmaz et al., 2013, *Staphylococcus aureus* and *Pseudomonas. aeruginosa*, Various Antibiotics

Yilmaz et al. [117], in separate experiments consisting of treatment of rat implant-associated in vitro-established infections, provide convincing evidence of phage anti-biofilm activity in the presence of antibiotic against one pathogen (*S. aureus* and phage Sb-1) but less robust results against a second (*P. aeruginosa* and phage vB_PsaP PAT14). Phages were applied directly to infections by injection, once per day for three days (10^7^ PFU/dose), and antibiotics were supplied intraperitoneally once per day for 14 days (20mg/kg teicoplanin for *S. aureus*; 120 mg/kg imipenem and cilastatin plus 25 mg/kg of amikacin for *P. aeruginosa*). With *S. aureus*, numbers were reduced relative to the untreated control by 7 logs given antibiotic-only treatment and by 9 logs with combination treatment (though only by 4 logs with treatment by phages alone). With *P. aeruginosa*, by contrast, 3 log reductions were seen with antibiotic-only treatment, but only 4 log reductions with either phage-only or combination treatments. These results might be consistent with phage virion production in vivo, though without volume or in situ phage titer information this possibility is not necessarily easily corroborated. The results nevertheless should be viewed as consistent with at least phage bactericidal activity particularly against *S. aureus*.

#### 4.3.4. Kaur et al., 2016, *Staphylococcus aureus*, linezolid

Kaur et al. [118] presents an in vivo testing, in mice, of prevention of *S. aureus* surface adherence to orthopedic-grade stainless steel Kirschner wires, i.e., as discussed above in terms of in vitro testing [109] (Section 4.2.5). Hydroxypropyl methylcellulose hydrogels impregnated with a combination of both phages and linezolid appear to have been slightly more effective at preventing bacterial colonization than impregnation with linezolid alone (4% *w*/*w* of wires), which in turn was slightly more effective than impregnation with phage MR-5 alone (10^9^ PFU/mL). Perhaps consistently, a faster drop off in phage titers in vivo was seen with antibiotic present versus without, but still with increases in titers in both cases early on during experiments. The in vivo results overall are suggestive of slightly lower phage activity in the absence of linezolid in comparison to what was observed in vitro [109]. Similar conclusions otherwise can be reached between the two studies, [118] and [109]. Therefore, as with the in vitro study, Kaur et al. [118] provides evidence of phage infection activity, but not necessarily substantial activity against bacteria which also have been substantially impacted by antibiotic.

#### 4.3.5. Oechslin et al., 2017, *Pseudomonas aeruginosa*, Ciprofloxacin

Oechslin et al. [103] explored the impact of a combination of a phage cocktail (dubbed PP1131 and delivered intravenously) and ciprofloxacin (also by injection) in a *P. aeruginosa* rat endocarditis model. After six hours of treatment, started 18 h post bacterial challenge, bacterial loads were reduced by 2–3 logs by either phage or antibiotic treatment, but by roughly 7 logs by combined phage and antibiotic treatment. Phage titers in plasma were approximately 10^8^ PFU/mL, but ~10^10^ PFU/g in association with aortic valve vegetations versus ~10^9^ CFU/g. Based on Poisson distributions (Section 2), it is difficult to say whether or not these results imply that phage production occurred in situ. This is because this is roughly a 10-fold excess of phages, which could easily give rise to 4-log reductions in bacterial densities by adsorption and resulting bactericidal effects alone. These results therefore are certainly consistent with phage bactericidal activity in the presence of ciprofloxacin but not necessarily with new phage production. On the other hand, the excess in phage numbers per gram of aortic valve vegetations relative to per mL of plasma, i.e., as much as a 100-fold excess, and as much as 1000-fold in excess relative to the phage-only control, are consistent with localized phage virion production in association with these localized high densities of bacteria.

Ciprofloxacin was dosed at 20 mg/kg with a single bolus at the same time as phages were applied and calculated MIC was 0.19 μg/mL. Given the short duration of the experiment as well as the substantial impact that antibiotic alone had on bacterial numbers, it seems reasonable to conclude that this proposed aortic valve vegetation-located phage replication occurred in association with bacteria that at least could have been exposed to 1× MIC or greater ciprofloxacin, as would be consistent with their in vitro, broth results presented in the same study (Section 4.1.8). These results, it should be noted and as the authors point out, may represent phage-antibiotic synergy as a 10^7^-fold reduction in bacterial numbers is in excess of 10^3^ × 10^3^ = 10^6^ approximate reduction as predicted for additive phage-antibiotic interactions.

#### 4.3.6. Vahedi et al., 2018, *Escherichia coli*, Ciprofloxacin

The results of Vahedi et al. [119], though positive for phage therapy impact on the treated *E. coli* mouse gastrointestinal colonization, neither support nor refute compatibility of phage activity with presence of ciprofloxacin. The phage used was not named and treatment doses were delivered orally by gavage.

#### 4.3.7. Summary of Evidence of Phage Infection Activity In Vivo in the Presence of Antibiotics

Animal experiments, as noted, are highly complex in terms of the state of the bacteria interacting with treatment phages. Therefore, the potential for phages to display infection activity while infecting bacteria that are not substantially affected by antibiotic presence, despite those bacteria being antibiotic susceptible, could be high. That results of these in vivo experiments are supportive of phage infection activity during treatments, and even in certain cases virion production, therefore might not be surprising. It is notable, however, that the more impressive of the animal co-treatment results (Section 4.3.5) is associated also with the most impressive of in vitro phage compatibility with antibiotic presence results (Section 4.1.8) [103]. Tentatively we might speculate that the complexity of treated environments may be an aid to phage compatibility with antibiotics, i.e., in terms of phages impacting especially those individual bacteria which, due to circumstances, are less antibiotic affected. This speculation certainly needs to be more rigorously explored, however.

### 4.4. Evidence of Phage-Infection Compatibility with Antibiotic Treatment, Conclusions

It is unfortunate that much of the strongest evidence of phage infection activity in the presence of antibiotics comes from the most complex experimental systems: in vitro biofilms or in vivo disease models. On the one hand, the results obtained suggest that there can be substantial latitude in terms of the potential for phages to kill bacteria and/or to produce new virions despite antibiotic presence. In particular, perhaps phages are infecting especially those bacteria which are least affected by antibiotic treatments. On the other hand, the ambiguities associated with the interpretation of many studies are highly suggestive of a utility to better characterizing phage compatibility with antibiotic treatments under more rigorous conditions, e.g., as via single-step growth experiments [90,91] as done in broth in which bacteria have been pre-treated by antibiotic prior to phage addition. Once so defined, it would be interesting to determine whether phage infection activity might in fact improve given greater experimental-system and bacterial heterogeneity during phage treatment in the presence of 1× MIC or higher antibiotic concentrations.

## 5. General Conclusions

Summers [122], in 2001, differentiated the history of phage therapy—at least in terms of the Western experience—into four periods, corresponding to “early enthusiasm, critical scepticism, abandonment, [and] recent interest and reappraisal”. The more modern of these periods arguably began, in the English-language literature, with the mouse study of Smith and Huggins [123] in 1983 and collections of clinical case studies published by Ślopek et al. from 1983 to 1987 [124,125,126,127,128,129,130]. From 1983 to 2016, nearly 40 human phage therapy studies were published, also in the English-language literature [2], including those of Ślopek et al. Many though by no means not all of these studies have involved co-treatment of phages with antibiotics (see also [7,131]), though it has not always been obvious how sensitive the treated bacteria were to the antibiotics being used. Historically, however, phage therapy has often been a response to failures of antibiotic treatment [3,6]. More recently, there also have been several case studies involving both phage therapy following antibiotic failures and continued antibiotic treatment even after phage therapy has begun [10,12,13,15,16,17]. Therefore, it would seem that the time has come for more rigorous scrutiny of phage activity as may or may not be present given antibiotic co-treatments of especially still antibiotic-susceptible bacteria.

The utility of phage-antibiotic co-treatments thus is being increasingly explored in the pre-clinical phage therapy literature [33,41]. Substantial evidence exists, however, that many antibiotics, when used at otherwise clinically relevant concentrations, particularly at or exceeding measured minimum inhibitory concentrations, can interfere with phage infection activity, and therefore with phage primary pharmacodynamic properties. Therefore, ideally phage infection capabilities in the presence of antibiotics, at those concentrations, would be assessed prior to proceeding to more complex, time-consuming, and expensive experimentation, such as animal testing. This idea of testing phages in vitro for basic infection activity prior to the start of more complex procedures is, of course, not a new concept to phage therapy. As noted by Eaton and Bayne-Jones in 1934 [132], p. 1773, “in vitro lysis of the infecting organism by the bacteriophage should always be demonstrated, preferably before the agent is used in treatment.” To fully assess phage infection activity, one may for example employ single-step growth-like experiments [90,91], looking for phage ability to lyse bacteria and produce new virions despite antibiotic presence.

Although phage virion production during phage therapy exists as an ideal, it nevertheless is possible for phages to kill bacteria without necessarily also producing phage progeny, resulting in what are known as abortive infections [42,43]. A complication, though, is that retention of phage bactericidal activity, particularly if absent phage-induced bacterial lysis, can be difficult to measure in the presence of antibiotics, since antibiotics also profoundly affect bacterial physiology. Therefore, assessment of the ability of antibiotic-susceptible bacteria to continue to replicate despite phage adsorption, given concurrent antibiotic exposure especially at or exceeding MIC, can be nonsensical as bacteria will continue to not replicate so long as antibiotic is present at sufficient concentrations. Removing antibiotic to observe phage impact in isolation, however, would represent a changing of conditions such that similarly we no longer can know whether phage bactericidal activity is present while antibiotic is still present. More sophisticated experiments therefore may be necessary to assess explicitly phage bactericidal activity, without associated bacteriolytic or virion production activities, in the presence of antibiotic.

Phages may primarily be used to target those bacterial subpopulations, in situ, which for whatever reason—e.g., poor vascularization, biofilm residence, decay in antibiotic densities over time—are exposed to lower concentrations of antibiotic [37,59]. In this case, phage-antibiotic compatibility may be less of a concern at least to the extent that antibiotics are able to eliminate one fraction of targeted bacteria while phages bactericidally eliminate the rest. This may be achieved particularly to the extent that phages are supplied in sufficient numbers that they can reach those bacteria that are less available to antibiotics without phages also being relied upon to display substantial in situ population growth. Above all, though to date we have a number of hints that 1× MIC or greater antibiotic concentrations will not necessarily completely inhibit phage infection-associated pharmacodynamic properties, perhaps particularly in more complex environments, that ability is not necessarily assured for a given phage, antibiotic, host bacterium, or treatment conditions. It can be useful, therefore, to confirm earlier rather than later during the development of phage-antibiotic co-treatment strategies, that reasonably robust phage antibacterial activity and/or phage virion production can occur in the presence of antibiotics that have been co-supplied at otherwise bacterial-infection inhibitory concentrations.

## Figures and Tables

**Table 1:** Studies suggesting inhibition of phage infection activity in the presence of antibiotics ^1,2^.

Subsection	Host	Phage	Antibiotic	GE	BC	BL	VP	LT	Ref
Section 3.1	*R. solanacearum*	various ^3^	rifampin				−		[68]
Section 3.1.1	*M. tuberculosis*	TM4	various ^4^	−					[69]
Section 3.1.2	*M. tuberculosis*	D29	various ^5^				−		[70]
Section 3.1.3	*M. tuberculosis*	D29	rifampin				−		[71]
Section 3.1.4	*M. tuberculosis*	D29	rifampin				−		[72]
Section 3.1.5	*M. tuberculosis*	D29	various ^6^				−		[73]
Section 3.2	*P. aeruginosa*	LUZ19	rifampin				−		[74]
Section 3.2.1	*B. subtilis*	SPO1	rifamycin			−	−		[75]
Section 3.2.2	*B. subtilis*	β22	rifampin				−		[76]
Section 3.2.3	*E. coli*	λvir	rifampin				−	−	[77]
Section 3.2.4	*E. coli*	T2	rifampin				−		[78]
Section 3.2.5	*E. coli*	T5	rifampin				−		[79]
Section 3.2.6	*E. coli*	Mu	rifampin				−		[80]
Section 3.2.7	*Pseudomonas*	PM2	rifampin				−		[81]
Section 3.2.8	*Pseudomonas*	various ^7^	rifampin				−		[82]
Section 3.3.1	*E. coli*	various ^8^	nalidixic acid				−		[83]
Section 3.3.2	*E. coli*	various ^9^	nalidixic acid				−		[84]
Section 3.3.3	*E. coli*	ECA2	various ^10^		−		−		[85]

^1^ Shown are approximate summaries of primary take-home messages of studies regarding phage activities in the presence of approximately 1× MIC concentrations of antibiotics or higher, where a ‘−‘ indicates a relative absence of demonstrated activity with most hosts tested, a ‘+’ a relative presence of activity, and ‘±’ ambiguous or multiple different results in terms of either phage gene expression (GE), phage bactericidal activity (BC), phage bacteriolytic activity (BL), or phage virion production (VP). LT stands for ‘lysis timing’ where a ‘−‘ in this column indicates lysis delay (thus, a ‘–‘ across the table tends to indicate negative impacts of antibiotic on phage infection activity). Note that for lytic phages, virion production implies bactericidal as well as bacteriolytic activity, though these properties are not indicated in the table unless they were explicitly measured in a study. ^2^ Please use this table as a guide-only to what studies are discussed in this review rather than as a definitive indication of phage properties in the presence of antibiotics, as actual results tend to be more complex than can be summarized this concisely. ^3^ Section 3.1, phages tested individually: ΦRSF1, ΦRSL1, ΦRSL2, and ΦRSB1. ^4^ Section 3.1.1, antibiotics tested individually: isoniazid, rifampin, or streptomycin. ^5^ Section 3.1.2, antibiotics tested individually: isoniazid or rifampin. ^6^ Section 3.1.5, antibiotics tested individually: amikacin, capreomycin, cycloserine, ethambutol, ethionamide, isoniazid, kanamycin, linezolid, moxifloxacin, ofloxacin, para-aminosalicylic acid, rifampin, or streptomycin. ^7^ Section 3.2.8, phages tested individually: PAK_P3 or PAK_P4. ^8^ Section 3.3.1, phages tested individually: 10, β, φR, φX174, T2 or T5. ^9^ Section 3.3.2, phages tested individually: φR, λ, T2, T4, or T5. ^10^ Section 3.3.3, antibiotics tested individually: chloramphenicol, ciprofloxacin, or tetracycline.

**Table 2:** Studies suggesting retention of phage infection activity in the presence of antibiotics^1,2.^

Subsection	Host	Phage	Antibiotic	BC	BL	VP	LT	CT	Ref
Section 4.1.1	*B. subtilis*	SPO1	nalidixic acid		+	+	−		[98]
Section 4.1.2	*B. subtilis*	various ^3^	nalidixic acid		+	+		?	[84]
Section 4.1.3	*B. subtilis*	SPP1	nalidixic acid		+		−	?	[99]
Section 4.1.4	*B. subtilis*	PBS2	nalidixic acid		+	−			[100]
Section 4.1.5	*M. aurum*	D29	various ^4^			±		?	[101]
Section 4.1.6	*B. subtilis*	AR9	rifampin			+		?	[102]
Section 4.1.7	*R. solanacearum*	various ^5^	rifampin			±		?	[68]
Section 4.1.8	*P. aeruginosa*	cocktail	various ^6^			+			[103]
Section 4.1.9	*A. baumannii*	KARL-1	various ^7^			+			[104]
Section 4.1.10	*E. coli*	ELY-1	ciprofloxacin			+			[105]
Section 4.2.1	*K. pneumoniae*	unnamed	amoxicillin			+		?	[106]
Section 4.2.2	*K. pneumoniae*	KPO1K2	ciprofloxacin			+		?	[107]
Section 4.2.3	*S. aureus*	SAP-26	various ^8^	+					[108]
Section 4.2.4	*E. coli*	T4	tobramycin			+		?	[39]
Section 4.2.4	*P. aeruginosa*	PB-1	tobramycin			−		?	[39]
Section 4.2.5	*S. aureus*	MR-5	linezolid	+				?	[109]
Section 4.2.6	*K. pneumoniae*	KPO1K2	amikacin			±		?	[110]
Section 4.2.7	*P. aeruginosa*	various ^9^	various ^10^			±		?	[18]
Section 4.2.8	*S. aureus*	SATA-8505	various ^11^			±		?	[111]
Section 4.2.9	*P. mirabilis*	vB_PmiS-TH	ampicillin	+					[112]
Section 4.2.10	*P. aeruginosa*	cocktail	ciprofloxacin	+				?	[113]
Section 4.2.11	*S. aureus*	PYO	various ^12^			+		?	[34]
Section 4.2.12	*P. aeruginosa*	various ^13^	ciprofloxacin			+		?	[114]
Section 4.3.1	*K. pneumoniae*	SS	amikacin			−		?	[115]
Section 4.3.2	*S. aureus*	MR-10	linezolid	−					[116]
Section 4.3.3	*S. aureus*	Sb-1	teicoplanin			+		?	[117]
Section 4.3.3	*P. aeruginosa*	vB_PsaP PAT14	various ^14^			+		?	[118]
Section 4.3.4	*S. aureus*	MR-5	linezolid			+		?	[118]
Section 4.3.5	*P. aeruginosa*	cocktail	ciprofloxacin			+		?	[103]
Section 4.3.6	*E. coli*	unnamed	ciprofloxacin						[119]

^1^ Shown are approximate summaries of primary take-home messages of studies regarding phage activities in the presence of approximately 1× MIC concentrations of antibiotics or higher. A ‘−‘ indicates a relative lack of phage infection activity (meaning a negative impact of antibiotic on phage infection a activity), a ‘+’ indicates relative presence of phage infection activity (relative, that is, to no or very little activity at all; thus a relative lack of highly substantial negative impact of antibiotic on phage infection activity is indicated with a ‘+’), and ‘±’ indicates multiple different results (some ‘+’ results in combination with some not strictly ‘+’ results, especially as when testing multiple phages or multiple antibiotics). Results are summarized in terms of phage bactericidal activity (BC), phage bacteriolytic activity (BL), and phage virion production (VP), or lysis timing (LT). For the latter, a ‘−‘ indicates lysis delay. Therefore, a mark of ‘–‘, across the table, tends to indicate negative impacts of antibiotic on phage infection activity. See the main text for relevant caveats regarding these summaries, as indicated in the column marked CT with a ‘?‘. That is, a ‘?’ indicates that relatively low negative impacts of antibiotics on phage infection activities as indicated by a given study may be somewhat debatable, though these caveats do not necessarily apply to all results provided by a given study, and nor have studies lacking an explicit indication of possible caveats necessarily either employed or indicated all possible relevant controls. Note that, for lytic phages, virion production implies bactericidal as well as bacteriolytic activity, though these properties are not indicated in the table unless they were explicitly measured in a study. Not presented in the table are the findings that phage φKZ of *P. aeruginosa* [74] and phage PBS2 of *B. subtilis* [89], both of which appear to be resistant to rifampin along with, for the latter, also other host RNA polymerase-inhibiting antibiotics, for both phages presumably as due to their apparently exclusive use of phage-carried, non-host RNA polymerase enzymes during infections (Section 3.2). ^2^ Generally, please use this table as a guide-only to what studies are discussed in this review rather than as a definitive indication of phage properties in the presence of antibiotics, as actual results tend to be more complex than can be summarized this concisely. ^3^ Section 4.1.2, phages tested individually: φ29, SP50, or SP82. ^4^ Section 4.1.5, antibiotics tested individually: clofazimine, colistin, dapsone, ethambutol, isoniazid, rifampin, or streptomycin. ^5^ Section 4.1.7, phages tested individually: ΦRP12 or ΦRP31, and also ΦRSB1, ΦRSL1, ΦRSL2, or ΦRPSF1. ^6^ Section 4.1.8, antibiotics tested individually: ciprofloxacin or meropenem. ^7^ Section 4.1.9, antibiotics tested individually: ciprofloxacin, colistin, or meropenem. ^8^ Section 4.2.3, antibiotics tested individually: azithromycin, rifampin, or vancomycin. ^9^ Section 4.2.7, phages tested individually: NP1 or NP3. ^10^ Section 4.2.7, antibiotics tested individually: ceftazidime, ciprofloxacin, colistin, gentamycin, or tobramycin. ^11^ Section 4.2.8, antibiotics tested individually: cefazolin, dicloxacillin, linezolid, tetracycline, or vancomycin. ^12^ Section 4.2.11, antibiotics tested individually: ciprofloxacin, daptomycin, erythromycin, gentamycin, linezolid, oxacillin, tetracycline, or vancomycin. ^13^ Section 4.2.12, phages tested individually: ΦPT-18[b], ΦPT-20[a], ΦPT-1S[a], ΦPT-5[a] or ΦPT-2[b]. ^14^ Section 4.3.3, antibiotics tested simultaneously: amikacin, cilastatin, and imipenem.

**Table 3:** Impact of Different Antibiotics on *S. aureus* Biofilm Support of Phage Virion Production **^1^**.

Antibiotic	Log Δ PFUs with 0× MIC	Log Δ PFUs with 2× MIC	Log Δ PFUs with 10× MIC
Ciprofloxacin	2	<2	0
Daptomycin	2	>1	1
Erythromycin	2	<1	−3
Gentamycin	2	0	−4
Linezolid	2	<1	−3
Oxacillin	2	2	1
Rifampin	2	<2	<0
Tetracycline	2	1	<0
Vancomycin	2	1	0

**^1^** Given simultaneous phage-antibiotic addition, as derived from the results of Dickey and Perrot [34], shown are estimated log change in phage titers within experiments (Log Δ PFUs). For example, 0 indicates no change, 2 indicates a 100-fold increase (thereby indicating phage population growth), −4 indicates a 10,000-fold decrease (the latter presumably because phages adsorbed to bacteria either immediately prior to or following bacterial death). For increased clarity, all numbers are approximations as obtained predominantly from their Figure 3. Roughly, values of 1 or more are indicative of at least moderately robust phage population growth.
